# Spectroscopic Evidence in Optical Absorption for the Formation and Transformation of Prenucleation Clusters of CdS Quantum Dots

**DOI:** 10.1021/acscentsci.6c00394

**Published:** 2026-06-29

**Authors:** Yang Li, Kui Yu, Yusha Yang, Andrei Sapelkin, Sanwei Guo, Xiaobo Zhu, Sijie Zhang, Xiaoqin Chen

**Affiliations:** † Engineering Research Center in Biomaterials, 12530 Sichuan University, Chengdu, Sichuan 610065, P. R. China; ‡ Department of Physics and Astronomy, 4617 Queen Mary University of London, 327 Mile End Road, London E1 4NS, United Kingdom; § Cannano Jiayuan (Guangzhou) Science & Technology Co. Ltd, Guangzhou 510700, P. R. China; ∥ Department of Physics, Guizhou University of Engineering Science, Bijie, Guizhou 551700, P. R. China

## Abstract

Prenucleation clusters (PNCs) that occupy a local minimum in the energy landscape have been suggested to form via chemical self-assembly prior to the nucleation and growth of colloidal semiconductor quantum dots (QDs) in reactions of individual cation and anion containing molecules. However, firm spectroscopic evidence for the PNC has previously proven elusive. Using two model reactions of cadmium oleate + 1-dodecanethiol and cadmium oleate + sulfur, we show the first signature signal of optical absorption between 260 and 290 nm for CdS PNCs. Controlling the PNC transformation into magic-size clusters (MSCs) in dispersion, we demonstrate a clear reciprocal relationship of the absorption strength between the PNC and the MSC. Our findings build a solid foundation for PNC formation in QD reactions and bring a deeper insight into the molecule–PNC–QD/MSC transition sequence, the knowledge of which enables the transformation of nanochemistry from an empirical art to a science.

## Graphical abstract

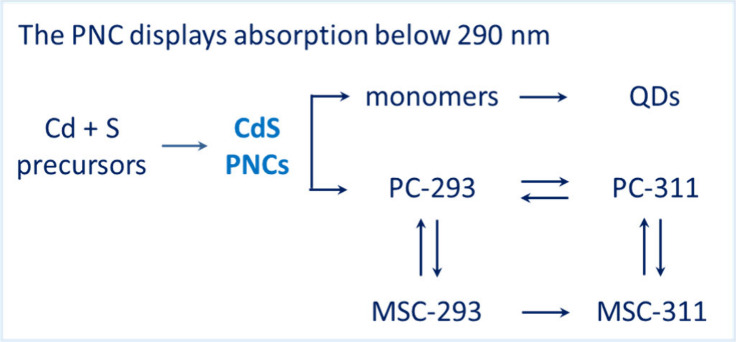

## Introduction

Crystallization from solutions has been essential in industry, including chemical and pharmaceutical manufacturing, and occurs ubiquitously in nature.^1–4^ In the explanation of the formation of solids from solutions, the one-step LaMer model universally dominated until as recent as 2004.^5–12^ Along with mounting evidence of prenucleation clusters (PNCs, with as yet unidentified compositions) in the field of ionic crystals,^13–17^ molecular crystals,^8,11,18–24^ and metallic crystals,^25–27^ two-step nucleation models are becoming popular progressively. These nonclassical models invoke the PNCs as intermediates on the way to the evolution of the solid.

For colloidal semiconductor quantum dots (QDs) of II–VI metal chalcogenide (ME) from chemical reactions of individual M and E containing molecules, a distinct two-step model based on the central role of the PNC has been put forward (Scheme 1a) based on the study of materials such as CdTe,^28^ ZnSe,^29^ CdSe,^30^ and ZnTe.^31^ This two-step model introduces a unique concept that in a reaction, a limited set of the M and E containing molecules undergoes chemical self-assembly,^4,32–36^ which results in the PNC (with M–E bonds) that occupies a local minimum in the energy landscape. The PNC forms as the intermediate (Steps 1a/1b) prior to the development of ME magic-size clusters (MSCs) (Step 2) and the nucleation and growth (N/G) of ME QDs via monomers (Mos) (Steps 2a/2b). Chemical self-assembly is a powerful tool in the construction of stable nanostructures that dwell in a local minimum of the energy landscape.^32^ When a group of molecules initially experiences physical self-assembly driven by noncovalent interactions, the repeated process of assembly and disassembly results in the formation of micellar-like assemblies that are at a local energy minimum (at the cost of increased entropy). Afterward, covalent bonds form inside each micellar-like assembly.^32^

**Scheme 1:**
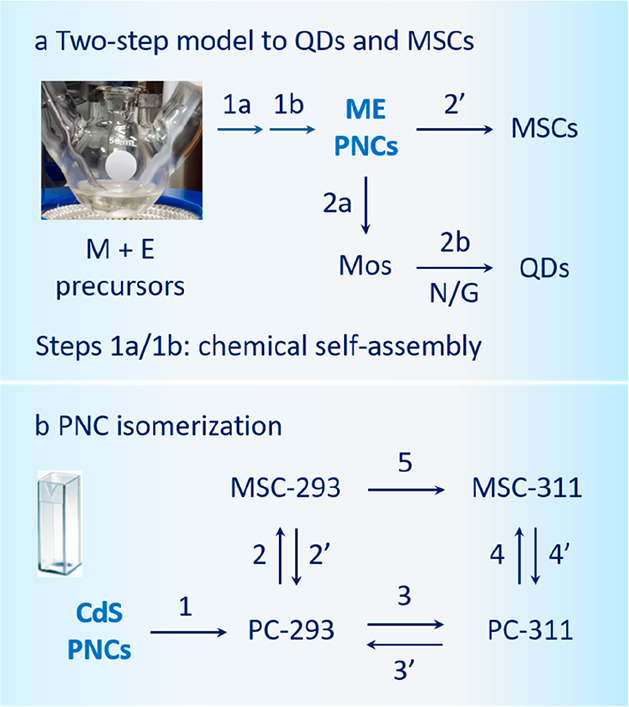
Pathways of the Formation and Transformation of ME PNCs (a) in Reactions of Individual M and E Containing Chemicals^a^ and (b) in Dispersion at Room Temperature^b^

a Chemical self-assembly (Steps 1a/1b) results in the PNC, which transforms into MSCs (Step 2) and QDs (Steps 2a/2b).
b The PNC in samples of CdS isomerizes to MSC-293 (Steps 1/2) and MSC- 311 (Steps 1/3/4). MSC-293 transforms to MSC-311 either directly (Step 5, featuring a continuous redshift) or indirectly (Steps 2′/3/4, exhibiting a stepwise redshift). MSC-311 transforms to MSC-293 indirectly (Steps 4′/3′/2, displaying a stepwise blueshift). The indirect transformations are assisted by PC-293 and PC-311, which are transparent at 293 and 311 nm, respectively, and at longer wavelengths.

The two-step model addresses the molecule–PNC–MSC/QD transition starting with chemical self-assembly (Scheme 1a).^28–31^ The formation of the PNC (Steps 1a/1b) requires a higher activation energy than that of the PNC–MSC isomerization (Step 2) and the PNC–QD transformation (Steps 2a/2b). The PNC-based transformations (Step 2 and Steps 2a/2b) feature the number conservation of the M–E bond and thus thermoneutrality of isodesmic reactions.^37–40^ Accordingly, Step 2 and Steps 2a/2b can occur in reactions at elevated temperatures and in dispersion under mild conditions. In dispersion, the PNC isomerizes into MSCs via the MSC precursor compounds (PCs). The PNC and the PC are transparent at the peak position of optical absorption of their corresponding MSCs and at longer wavelengths.^28,31^ Table S1 provides additional information for the PNC, PC, MSC, and monomer (Mo).

There is mounting evidence of the PNC from QD reactions of CdS,^41–45^ CdSe,^30,45–48^ CdTe,^28,31,49–52^ ZnS,^45,53^ ZnSe,^29,45,54–57^ and ZnTe.^31,58^ Currently, however, the QD field embraces mainly the one-step LaMer model^5–7,12,59^ rather than the two-step model (Scheme 1a).^28–31^ We see the core of the problem in the lack of spectroscopic evidence of the PNC; the scientific advance of colloidal chemistry of semiconductor MSCs and QDs experiences its vicissitudes.^60^

Here, we report the first spectroscopic evidence that the PNC displays a broad signal of optical absorption between 260 and 290 nm, using CdS as a model system with two reactions studied, cadmium oleate (Cd­(OA)_2_) + 1-dodecanethiol (CH_3_(CH_2_)_11_–SH) in 1-octadecene (ODE) as well as Cd­(OA)_2_ + sulfur (S) in ODE. Cd­(OA)_2_ has optical absorption peaking at 270 and 280 nm.^61^ When the PNC forms (Scheme 1a Steps 1a/1b) in the two reactions prior to the N/G of CdS QDs, the absorption strength between 260 and 290 nm increases unambiguously. Scheme 1b reveals the pathway of the PNC–MSC isomerization when a sample of CdS (containing the PNC) is dispersed. The development of MSC-293 (with optical absorption peaking at ∼293 nm via Scheme 1b Steps 1/2) and MSC-311 (with optical absorption peaking at ∼311 nm via Scheme 1b Steps 1/3/4) is accompanied by the reduction of the absorption strength between 260 and 290 nm. This provides further support for the PNC presence in the sample. An isosbestic point at ∼288 nm is seen. CdS MSC-293 has not been reported previously (Table S2). Also, we show that MSC-293 transforms to MSC-311 directly (Scheme 1b Step 5), displaying a continuous redshift of optical absorption from ∼293 to ∼311 nm, as well as indirectly (Scheme 1b Steps 2′/3/4) with a stepwise redshift that the strength at 293 and 311 nm monotonically diminishes and increases, respectively. Indirectly (Scheme 1b Steps 4′/3′/2), MSC-311 transforms to MSC-293 with a stepwise blueshift that the strength at 311 and 293 nm monotonically reduces and enhances, respectively. For the two indirect transformations that are assisted by the PC, they feature an isosbestic point at ∼300 nm with little change of the absorption strength between 260 and 290 nm.

The present study brings firm evidence in optical absorption for the PNC addressed in the two-step model with the molecule–PNC–QD/MSC transition sequence presumed (Scheme 1a).^28–31^ The PNC can be a powerful tool in the arsenal of colloidal chemistry. That the absorption strength below 290 nm is larger than that of a Cd precursor due to the CdS PNC formation builds a solid foundation on which the PNC formation can be monitored (Figure SA).^62^ With the discovery of MSC-293 and its direct isomerization to MSC-311, it is now easy to understand the unexplained appearance of CdS MSCs with the first exciton absorption peak at 305 nm instead of at 311 nm (Figure SB).^61^

## Results and Discussion

To obtain spectroscopic evidence of the PNC formation in reactions and the PNC transformation in dispersion, we used CdS as a model system and studied two reactions with a heating-up approach. One reaction was Cd­(OA)_2_ + C_12_H_25_–SH in ODE (Figures 1–4), and the other reaction was Cd­(OA)_2_ + sulfur (S) in ODE (Figures 5 and 6). Each reaction had a feed molar ratio of 4Cd–1S, a feed S concentration of ∼30 mmol kg^–1^, and a total weight of ∼5.0 g. The formation of the PNC (Scheme 1a Steps 1a/1b) in the two reactions was above 200 °C (Figure 1) and 160 °C (Figure 5), respectively, as indicated by the increase of absorption strength between 260 and 290 nm.

**Figure 1:**
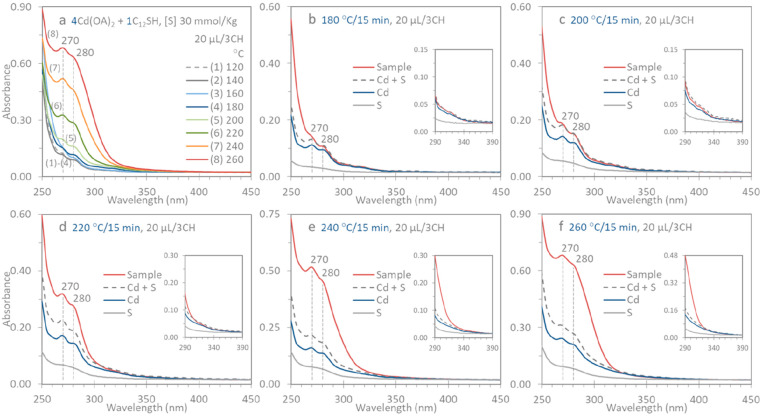
Development of the CdS PNC above 200 °C in the reaction of Cd­(OA)_2_ and C_12_H_25_–SH in ODE. During a heating-up process with a step of 20 °C, samples were extracted after 15 min at each step. For the measurement of optical absorption, an aliquot (20 μL) of each sample was placed in 3.0 mL of CH (3CH) (a). Prior to the N/G of QDs, the absorption increased between 260 and 290 nm when the temperature was raised from 200 °C (trace 5) to 260 °C (trace 8). The sample absorption (burgundy traces) is compared with the superimposed signal (dashed traces) of Cd­(OA)_2_ (blue traces, with peaks at 270 and 280 nm) and C_12_H_25_–SH (gray traces) (b–f). Each inset shows the conventional measurement from 290 nm to longer wavelengths. Between 260 and 290 nm, the difference between the burgundy and dashed traces widened from Sample 220 °C (d) to Sample 260 °C (f) via Sample 240 °C (e). The continuous increase of the sample absorption indicates the incessant formation of the PNC from 220 to 260 °C (Scheme 1a Steps 1a/1b). The absorption difference was due to the presence of the PNC (Scheme 1a Steps 1a/1b), and the difference widening was associated with the incessant formation of the PNC. The strength of the burgundy traces increased from 200 °C (c) to 260 °C (f).

When a sample (20 μL, with the PNC) was dispersed in a mixture of cyclohexane (CH, 3.0 mL) and methanol (MeOH, 10 μL) (CH–MeOH), MSC-293 (Scheme 1b Steps 1/2) and/or MSC-311 (Scheme 1b Steps 1/3/4) appeared (panels a–c of Figures 2 and 6); meanwhile, the absorption strength between 260 and 290 nm decreased due to the consumption of the PNC. Directly, MSC-293 transformed to MSC-311 (Scheme 1b Step 5), displaying a continuous redshift from ∼293 to ∼311 nm (panels e and d of Figures 2 and 6). The presentation format is similar for Figures 2 and 6. Indirectly, MSC-293 transformed to MSC-311 (Scheme 1b Steps 2′/3/4) exhibiting a stepwise redshift (Figure 3), and MSC-311 transformed to MSC-293 (Scheme 1b Steps 4′/3′/2) with a stepwise blueshift (Figure 4). These two PC-assisted transformations featured an isosbestic point at ∼300 nm and little change of the absorption strength between 260 and 290 nm.

**Figure 2:**
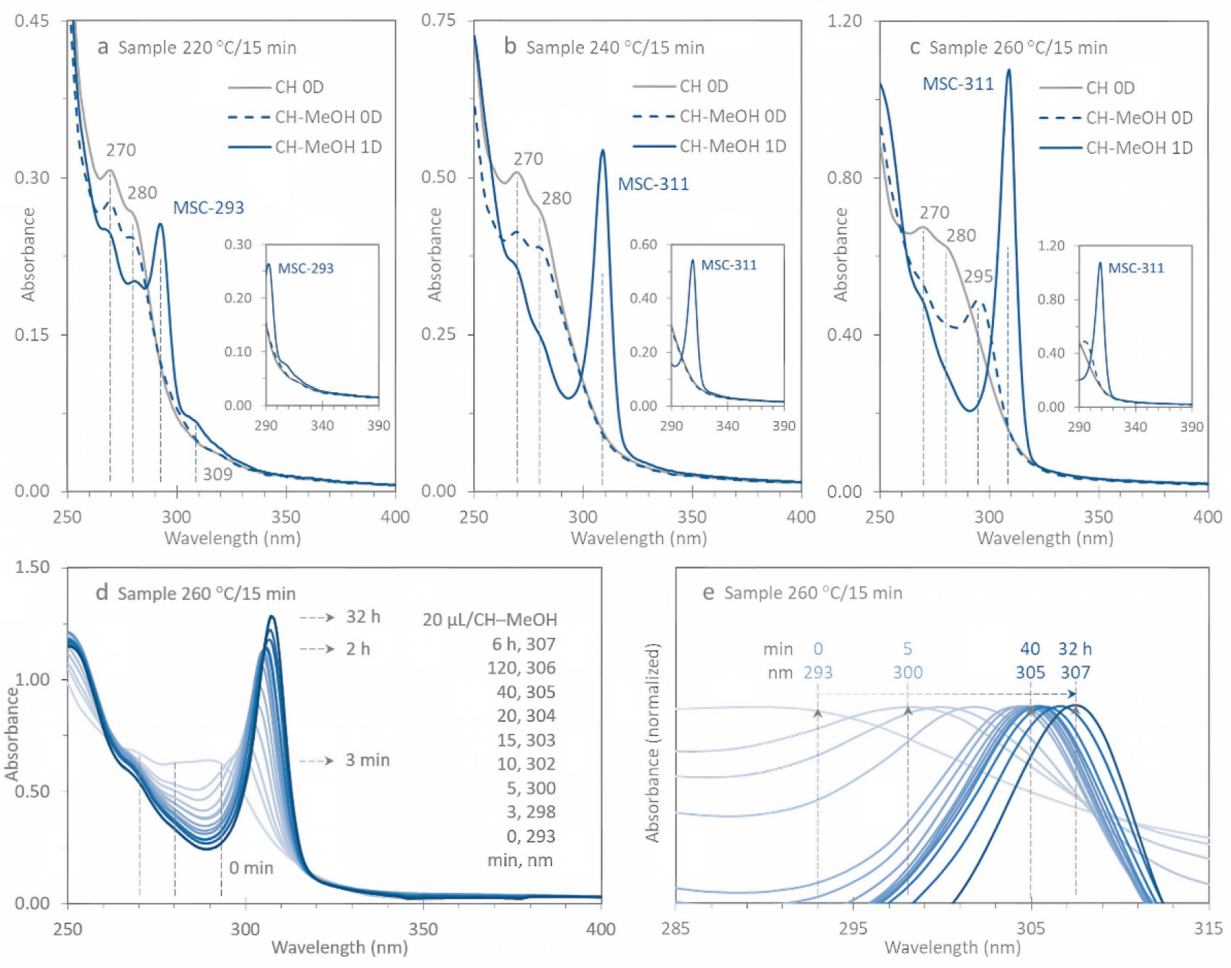
Isomerization from the CdS PNC to MSCs in dispersion at room temperature. The three samples in the top panels (a–c) were from the Figure 1 reaction batch. The sample in the bottom panels (d) and (e) was from another batch. For the measurement of optical absorption (a–c), an aliquot (20 μL) of each sample was placed in CH (0D gray traces) as well as in CH–MeOH (0D dashed traces and 1D blue traces). Each inset highlights the conventional measurement from 290 nm to longer wavelengths. For (d) and (e), the sample (20 μL) was placed in CH–MeOH, and a continuous redshift from 293 to 307 was seen over 32 h. In (e) (normalized), the spectra collected at 0, 5, and 40 min are indicated, together with that at 32 h. When MSC-293 and MSC-311 developed, the absorption strength between 260 and 290 nm decreased.

**Figure 3:**
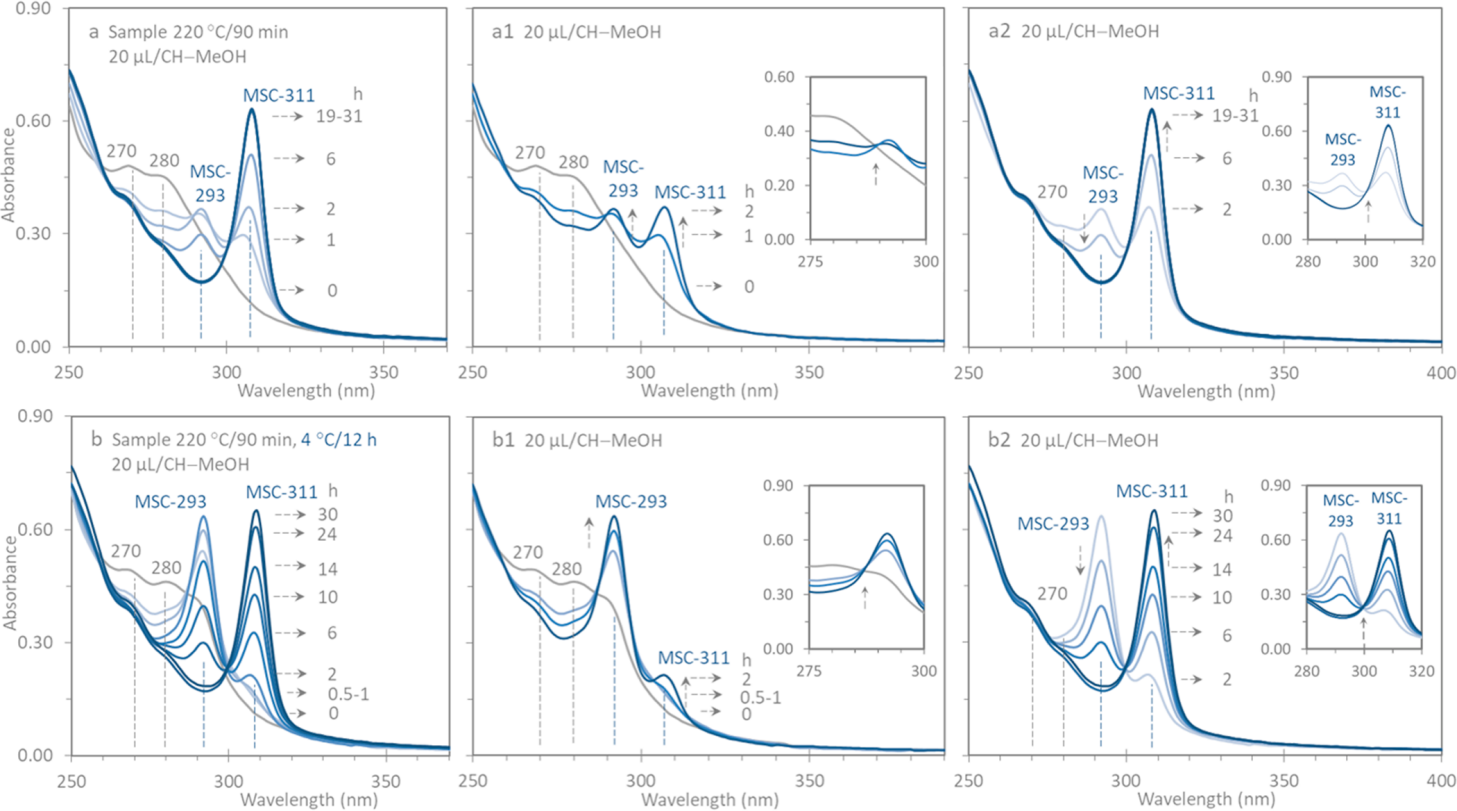
Isomerization from the CdS PNC to MSCs in dispersion with evidence of isosbestic points. A Figure 1 reaction was kept at 220 °C, and a sample was taken after 90 min. A portion of the sample was stored at 4 °C for 12 h. For the measurement of optical absorption, an aliquot (20 μL) of the sample (before and after storage) was dispersed in CH–MeOH. For the as-synthesized sample (a), eight spectra were obtained within 31 h; they are presented in panels (a1) and (a2). Panel (a1) has the first three spectra (0–2 h), and panel (a2) has six spectra (2–31 h). For the incubated sample (b), nine spectra were obtained within 30 h; they are shown in panels (b1) and (b2). Panel (b1) has the first four spectra (0–2 h), and panel (b2) has six spectra (2–30 h). It is noteworthy that the strength between 260 and 290 nm decreased in (a1) and (b1) but barely changed in (a2) and (b2). The isosbestic points are highlighted by the four insets.

**Figure 4:**
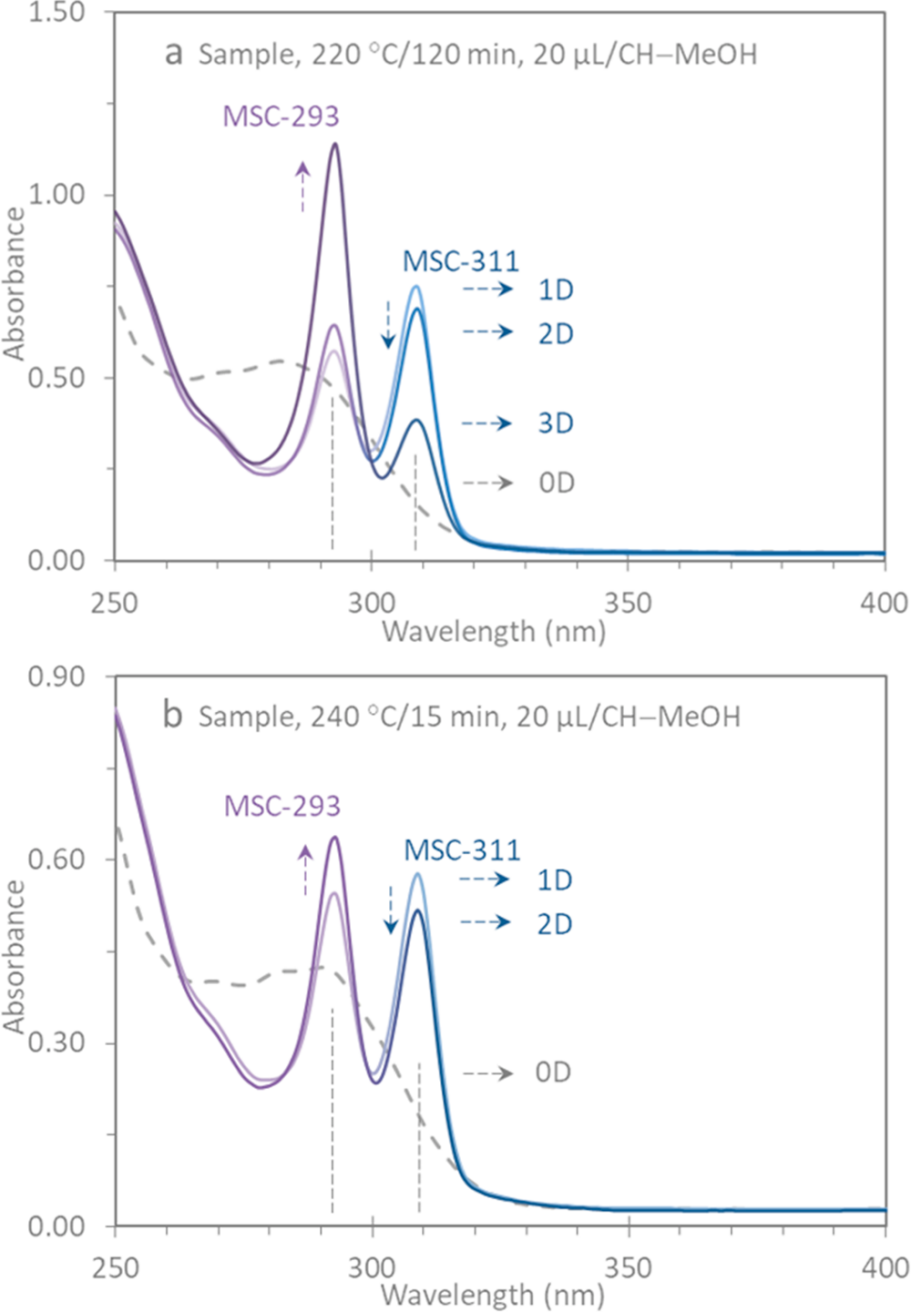
Isomerization from MSC-311 to MSC-293 in dispersion at room temperature. Two samples were studied. One sample (220 °C/120 min) was from the Figure 3 reaction but a different batch (a). The other sample (240 °C/15 min) was from the Figure 1 reaction but a different batch (b). For the measurement of optical absorption, an aliquot (20 μL) of each sample was placed in CH–MeOH; the dashed traces were obtained immediately (0D). At 1 day, both MSC-311 and MSC-293 developed. From 1 to 3 days (a) and from 1 to 2 days (b), MSC-311 decreased while MSC-293 increased (Scheme 1b Steps 4′/3′/2) at the same time.

### The PNC Formation in the Reaction of Cd­(OA)_2_ and C_12_H_25_–SH

In Figure 1, we show that the CdS PNC formed above 200 °C in the reaction of Cd­(OA)_2_ and C_12_H_25_–SH. The reaction temperature increased from 60 to 260 °C with a step of 20 °C, and 11 samples were extracted at each step after 15 min. For the measurement of optical absorption, an aliquot (20 μL) of each sample was placed in 3.0 mL of CH (3CH). Panel a of Figure 1 has eight spectra of samples taken at 120–260 °C. The strength between 260 and 290 nm barely changed for Samples 120–180 °C (traces 1–4), while it increased significantly for Samples 200–260 °C (traces 5–8). The flat background in the range of 340–450 nm (traces 1–8) indicates that no QDs formed. In Figure S1-1, we show that the N/G of QDs occurred above 260 °C.

We thus conclude that prior to the N/G of QDs (panel a of Figure 1), the increase of the absorption strength between 260 and 290 nm indicates the formation of the PNC with the Cd–S bond; the larger the strength is, the more the PNC forms. To further validate these findings, we compared the absorption of the samples (burgundy traces) with the superimposed signal (dashed traces) of Cd­(OA)_2_ (blue traces, with peaks at 270 and 280 nm) and C_12_H_25_–SH (gray traces) (panels b–f of Figure 1). The blue traces were from a Cd­(OA)_2_ batch with a concentration of 120 mmol kg^–1^ in ODE (without C_12_H_25_–SH). The gray traces were from a C_12_H_25_–SH batch with a concentration of 30 mmol kg^–1^ in ODE (without Cd­(OA)_2_). Similar to the Figure 1a reaction, the two batches had a weight of ∼5.0 g, and sampling was performed during the temperature increase from 60 to 260 °C with a step of 20 °C and an elapse time of 15 min at each step.

For samples taken at 180 °C (Figure 1b) and 200 °C (Figure 1c), the strength difference between the burgundy and the corresponding dashed traces is small in the range of 260 to 290 nm. However, for samples taken at 220 °C (Figure 1d) to 260 °C (Figure 1f) through 240 °C (Figure 1e), the difference increased. Hence, the continuous increase of the sample absorption (from traces 5–8 of Figure 1a) indicates the incessant formation of the PNC from 220 to 260 °C (Scheme 1a Steps 1a/1b). In a side note, the conventional measurement usually starts from 290 nm to longer wavelengths (insets in panels b–f of Figure 1). Therefore, the absorption change in the range of 250 to 290 nm could be overlooked for the study of PNC formation.^41–45^ With a similar presentation format to that of panels b–f of Figure 1, Figure S1-2A presents the comparison of optical absorption of samples obtained at 60 to 160 °C. These samples barely had the PNC. Therefore, the strength difference is small for the burgundy and corresponding dashed traces in the range of 260 to 290 nm. At 220 °C, the PNC formed (Figure S1-2B). In Figure S1-3A, we present the spectra of Figure 1 after the subtraction of the background; indeed, the broad signal of optical absorption between 260 and 290 nm increased apparently when the temperature was above 200 °C. In Figure S1-3B, we show a numerical analysis to further endorse the PNC formation.

### The PNC Transformation in Dispersion

The full set of spectra of the 11 samples from the Figure 1a reaction in CH (0D) is shown in panel a of Figure S1-4. After 1 day (1D), spectra were collected again and are presented in panel b of Figure S1-4. No MSCs developed. MeOH promotes the PNC–MSC isomerization.^41,42,63–66^ To study the PNC–MSC transformation in dispersion, an aliquot (20 μL) of each of the 11 samples was also dispersed in CH–MeOH. Spectra of optical absorption were collected immediately (0D, panel c of Figure S1-4) and after 1 day (1D, panel d of Figure S1-4). MSC-293 and MSC-311 were seen. To gain a better idea of the PNC transformation in dispersion, the spectra in Figure S1-4 are reproduced in panels a–c of Figure 2, Figure S2-1, and Figure S2-2. The first two figures deal with three prenucleation-stage samples (with the PNC) extracted at 220 to 260 °C, while the last figure shows another three samples (without the PNC) obtained at 160 to 200 °C.

In the top panels of Figure 2, each of the Samples 220 °C (a), 240 °C (b), and 260 °C (c) has three spectra with gray traces from CH (0D), dashed traces from CH–MeOH (0D), and blue traces from CH–MeOH (1D). In CH–MeOH (dashed traces), MSC-293 was seen (c); after 1 day (blue traces), MSC-293 (a) and MSC-311 (b and c) were seen. We suggest that the PNC isomerized mostly to MSC-293 (a) (Scheme 1b Steps 1/2) and MSC-311 (b and c) (Scheme 1b Steps 1/3/4).

During the PNC–PC/MSC transformation in dispersion, the absorption strength between 260 and 290 nm decreased clearly. This is consistent with the above interpretation because when the PNC formed in the reaction (traces 5–8, Figure 1a), the absorption strength between 260 and 290 nm increased. When a sample with the PNC was dispersed (panels a–c of Figure 2), the absorption strength between 260 and 290 nm was larger in CH (gray traces) than in CH–MeOH (dashed traces). Furthermore, along with the evolution of the MSCs in CH–MeOH, the strength decreased simultaneously at 270 and 280 nm (from the dashed to blue traces). From Sample 220 °C (Figure 2a) to Sample 260 °C (Figure 2c), the amount of the MSC increased (blue traces). This agrees with the increase of the PNC amount from Sample 220 °C to Sample 260 °C, as indicated by the enhancement of the absorption strength between 260 and 290 nm (gray traces from a to c). The more the PNC forms, the more the MSC develops.

To further examine the development of MSC-311 in CH–MeOH, we monitored *in situ* the evolution of optical absorption of Sample 260 °C/15 min (the bottom panels of Figure 2). In a side note, MSC-311 seems to develop more readily than MSC-293 when a relatively late-stage sample is placed in CH–MeOH. Sample 260 °C/15 min was from a different batch; the temperature of a Figure 1 reaction was kept at 240 °C for 15 min and then at 260 °C for 15 min. For the measurement, an aliquot (20 μL) of the sample was placed in CH–MeOH. Within 32 h, 23 spectra were obtained (Figure S2-3); Figure 2d shows 15 of them, with the continuous redshift from ∼293 to 307 nm highlighted in Figure 2e (normalized). It is of help to point out that a continuous redshift of optical absorption has been observed during isomerization from CdS MSC-345 to MSC-360,^44^ from CdTe MSC-448 to MSC-488,^64^ and from CdTeSe MSC-399 to MSC-422.^65^

For the development of MSC-311, when a sample is in dispersion of CH–MeOH, there are two probable pathways. One pathway is the PNC to MSC-311 via PC-293 and PC-311 (Scheme 1b Steps 1/3/4). The other pathway is the PNC to MSC-311 via PC-293 and MSC-293 (Scheme 1b Steps 1/2/5). When MSC-293 transforms directly, the intermediates display measurable optical absorption; thus, an uninterrupted redshift of optical absorption from 293 to 311 nm is seen. The two pathways can proceed at the same time.

In Figure 3, we show the appearance of isosbestic points (at ∼288 and ∼300 nm) during the PNC–MSC isomerization in dispersion at room temperature. When the isosbestic points at 288 and 300 nm appeared, the absorption strength between 260 and 290 nm decreased and changed little, respectively. A Figure 1a reaction was kept at 220 °C, and a sample was taken after 90 min. For the measurement of optical absorption, an aliquot (20 μL) of Sample 220 °C/90 min (before and after storage at 4 °C for 12 h) was dispersed in CH–MeOH. For the as-synthesized sample (the top panels of Figure 3), eight spectra were obtained within 31 h (panel a); they are presented in panels a1 and a2. Panel a1 has the first three spectra (0–2 h), and Panel a2 has six spectra (2–31 h). For the incubated sample (the bottom panels of Figure 3), nine spectra were obtained within 30 h (panel b); they are shown in panels b1 and b2. Panel b1 has the first four spectra (0–2 h). Panel b2 has six spectra (2–30 h).

In the first 2 h in dispersion (Figure 3a1,b1), the strength around 270–280 nm decreased and that at ∼293 and ∼310 nm increased concurrently. An isosbestic point was located at ∼288 nm (insets). The strength reduction between 260 and 290 nm is attributed to the consumption of the PNC. The strength intensification at 293 and 310 nm is associated with the development of MSC-293 and MSC-311, respectively. Isomerization from the PNC to MSC-293 (Scheme 1b Steps 1/2) and MSC-311 (Scheme 1b Steps 1/3/4) dominated in this period.

In the following hours (Figure 3a2,b2), the strength at ∼293 nm decreased monotonically, which coincided with the monotonic increase of the strength at ∼310 nm. MSC-311 appeared in a single-ensemble form without the coexistence of MSC-293 at 19 h and after (a2) as well as at 24 h and after (b2). An isosbestic point was observed at ∼300 nm (insets). Isomerization from MSC-293 to MSC-311 took over, which was assisted by the PC (Scheme 1b Steps 2′/3/4). The strength in the range of 250–270 nm remained almost intact, indicating little change of the PNC in this period.

When Sample 220 °C/90 min (with or without storage) was dispersed in CH–MeOH, isomerization from the PNC to MSC-293 (Scheme 1b Steps 1/2) and from the PNC to MSC-311 (Scheme 1b Steps 1/3/4) dominated first (Figure 3a1,b1 within 2 h), followed by PC-assisted isomerization from MSC-293 to MSC-311 (Scheme 1b Steps 2′/3/4) (Figure 3a2,b2 after 2 h). When the PNC transformed to MSC-293 and MSC-311, the absorption at 270 and 280 decreased, together with an isosbestic point at ∼288 nm (panels a1 and b1). When MSC-293 transformed to MSC-311, the strength in the range of 250–275 nm changed little, together with an isosbestic point at ∼300 nm (panels a2 and b2). The PNC involved little in the MSC-293 to MSC-311 transformation. Similar results were obtained when Sample 220 °C/60 min was placed in CH–MeOH (Figure S3-1 with a similar presentation formation to that of Figure 3). Figure S3-2 presents the absorption spectra of Sample 220 °C/120 min and Sample 220 °C/150 min in CH–MeOH. Within 1 h, MSC-311 developed. Again, MSC-311 is more ready to show up (than MSC-293) when a relatively late-stage sample is placed in CH–MeOH.

In Figure 4, we show the PC-assisted isomerization from MSC-311 to MSC-293 in dispersion at room temperature. Sample 220 °C/120 min (a) was from the Figure 3 reaction but a different batch. Sample 240 °C/15 min (b) was from the Figure 1 reaction but a different batch. For optical absorption measurements, each sample (20 μL) was added to CH–MeOH. The dashed traces correspond to the spectra recorded immediately (0D). For the spectra collected at 1D and after, they are represented by solid traces with the color purple for MSC-293 and blue for MSC-311.

When the two samples were dispersed (dashed 0D traces), no sharp peaks at ∼293 and ∼311 nm were seen. At 1 day, the development of both MSC-293 and MSC-311 was seen (solid 1D traces). This was due to isomerization from the PNC to MSC-293 and to MSC-311, with the pathway of Scheme 1b Steps 1/2 and Scheme 1b Steps 1/3/4, respectively. The strength between 260 and 290 nm reduced clearly. After 1 day (solid 2D and 3D traces), MSC-311 decreased while MSC-293 increased simultaneously. The strength in the range of 250–275 nm hardly changed. Thus, the pathway of the MSC-311 to MSC-293 isomerization underwent Scheme 1b Steps 4′/3′/2, with little involvement of the PNC. The reversible isomerization between MSC-293 and MSC-311 is noteworthy.

To study the PC-assisted isomerization from MSC-311 to MSC-293, a relatively late-stage sample without storage is preferred. When the Figure 4b sample (240 °C/15 min) was stored at 4 °C for 2 days and then dispersed in CH–MeOH, MSC-293 and MSC-311 developed within 3 h, followed by the PC-assisted isomerization from MSC-293 to MSC-311 (Figure S4-1). The strength in the range of 250–275 nm barely changed during the isomerization (Scheme 1b Steps 2′/3/4). Low-temperature storage favors the isomerization from the PNC to PC/MSC-293 (Scheme 1b Steps 1/2) more than to PC/MSC-311 (Figure S4-2) (Scheme 1b Steps 1/3/4).

### The PNC Formation in the Reaction of Cd­(OA)_2_ and S

In Figure 5, we show that the PNC evolved above 160 °C in the reaction of Cd­(OA)_2_ and S in ODE. The presentation format is similar to that of Figure 1. In total, eight samples were obtained when the reaction temperature increased from 80 to 220 °C with a step of 20 °C and an elapse time of 15 min at each step. For the measurement of optical absorption, an aliquot (20 μL) of each sample was placed in 3.0 mL of CH (3CH) (panel a of Figure S5-1). Panel a of Figure 5 has six spectra of samples taken at 120–220 °C (traces 1–6). The absorption between 260 and 290 nm changed little for Sample 120 °C (trace 1) and Sample 140 °C (trace 2), while it increased considerably for Sample 160 °C (trace 3) to Sample 200 °C (trace 5) via Sample 180 °C (trace 4). The strength between 260 and 290 nm was kept almost intact for Sample 200 °C (trace 5) and Sample 220 °C (trace 6). QD-382 and QD-400 were seen in the last two samples, respectively.

**Figure 5:**
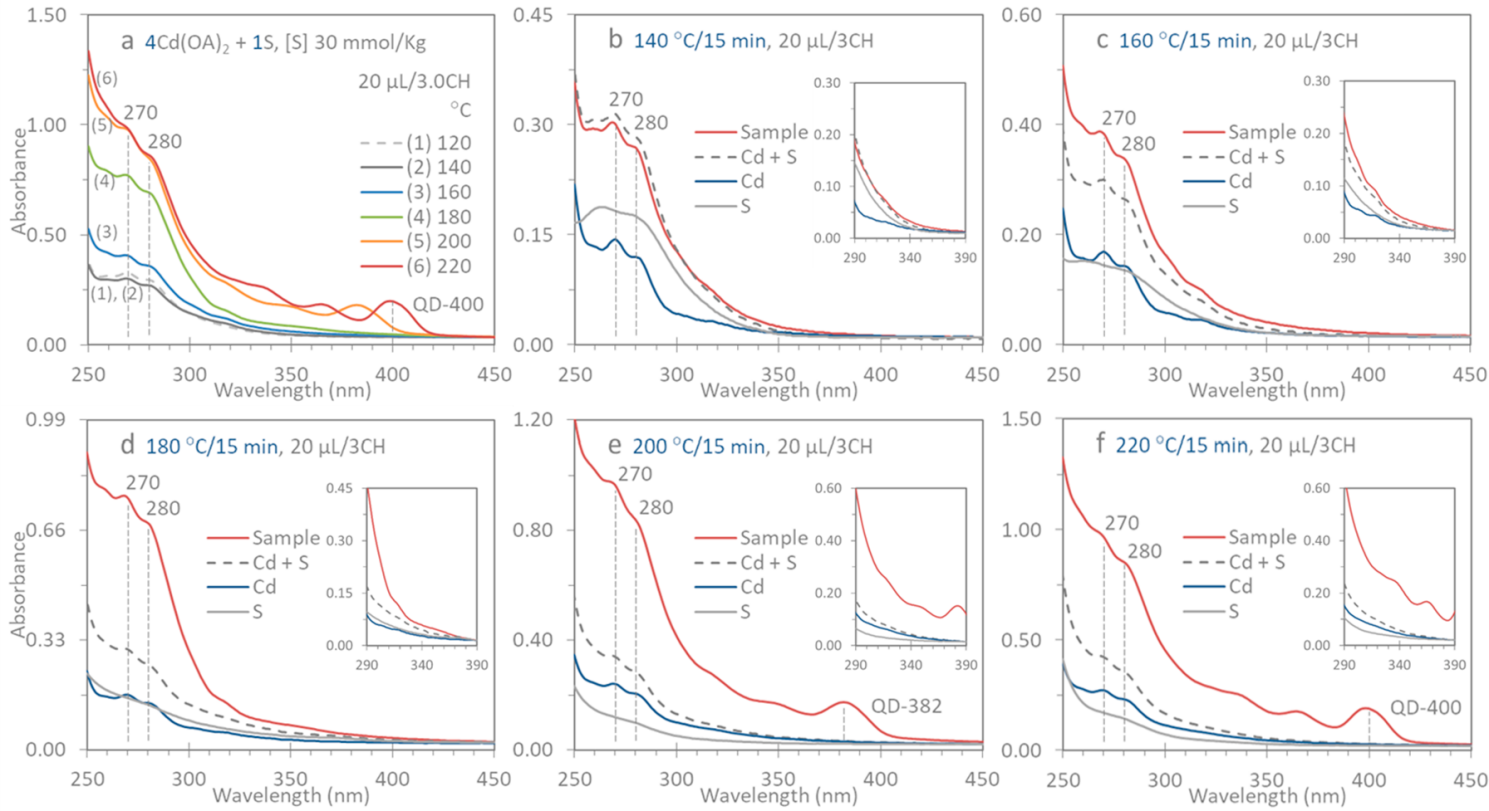
Development of the CdS PNC above 160 °C in the reaction of Cd­(OA)_2_ and S in ODE. The presentation format is similar to that of Figure 1. During a heating-up process with a step of 20 °C, samples were taken after 15 min at each step. For the measurement of optical absorption, an aliquot (20 μL) of each sample was placed in 3CH. (a) Between 260 and 290 nm in particular, the absorption increased when the temperature was raised from 160 °C (trace 3) to 200 °C (trace 5) through 180 °C (trace 4); the absorption changed little from traces 5 to 6. At 200 °C, CdS QD-382 appeared and grew to QD-400 at 220 °C. The incessant development of the PNC above 160 °C is supported by panels (b–f). Each inset features the conventional measurement from 290 nm to longer wavelengths.

Accordingly, prior to the N/G of QDs in the reaction, the absorption between 260 and 290 nm increased at 160 °C (trace 3) and 180 °C (trace 4). We attribute the increase to the formation of the PNC with the Cd–S bond; the larger the strength between 260 and 290 nm is, the more the PNC forms. In the initial stage of the N/G of QDs at 200 °C (trace 5), the absorption between 260 and 290 nm increased too. The PNC continuously forms (via Scheme 1a Steps 1a/1b) and transforms into QDs (via Scheme 1a Steps 2a/2b). A QD sample can have the PNC,^28–30,41,42,49,54,56^ as shown by Figures S1-1 to S5-3.

Similarly, we compared the absorption of the samples (burgundy traces) with the superimposed signal (dashed traces) of Cd­(OA)_2_ (blue traces, with peaks at 270 and 280 nm) and S (gray traces) (panels b–f of Figure 5). The blue traces were from a Cd­(OA)_2_ batch with a concentration of 120 mmol kg^–1^ in ODE (without S). The gray traces were from a S batch with a concentration of 30 mmol kg^–1^ in ODE (without Cd­(OA)_2_). The two batches had a similar weight of ∼5.0 g, and sampling was performed in a similar way during the temperature increase from 80 to 220 °C with a step of 20 °C and an elapse time of 15 min at each step.

With a similar presentation format to that of panels b–f of Figure 5, panels b–d of Figure S5-1 present the comparison of the optical absorption of samples obtained at 80 to 120 °C. These three samples had little PNC. Thus, the difference of the absorption strength in the range of 260 to 290 nm is little between the burgundy and the corresponding dashed traces. For samples taken at 140 °C (Figure 5b), the strength difference is little too. However, for samples taken at 160 °C (Figure 5c) to 220 °C (Figure 5f) via 180 °C (Figure 5d) and 200 °C (Figure 5e), the strength difference increased. Hence, the continuous increase of the sample absorption indicates the incessant formation of the PNC from 160 to 200 °C (Scheme 1a Steps 1a/1b). In a side note, the conventional measurement usually starts from 290 nm to longer wavelengths (insets in panels b–f of Figure 5). Previous studies of PNC formation perfectly overlooked the absorption change in the range of 250 to 290 nm.^41–45^ In Figure S5-2, we show that when the temperature was above 160 °C, the PNC indeed formed with the broad signal of optical absorption between 260 and 290 nm.

### The PNC Transformation in Dispersion

We repeated the Figure 5 reaction, with eight samples obtained at 80–220 °C. For the measurement of optical absorption, an aliquot (20 μL) of each sample was placed in CH and in CH–MeOH. From each dispersion, two spectra were collected immediately (0D) and after 1 day (1D). In Figure S5-3, the two sets of spectra from CH are shown in panels a (0D) and b (1D), and those from CH–MeOH are shown in panels c (0D) and d (1D). To gain a better idea of the transformation of the PNC in dispersion, the spectra in Figure S5-3 are replicated in panels a–c of Figure 6, Figure S6-1, and Figure S6-2. The first two figures show the three samples extracted at 160 to 200 °C, while the last figure shows another three samples obtained at 100 to 140 °C. Samples 160 to 200 °C had the PNC, while Samples 100 to 140 °C did not.

**Figure 6:**
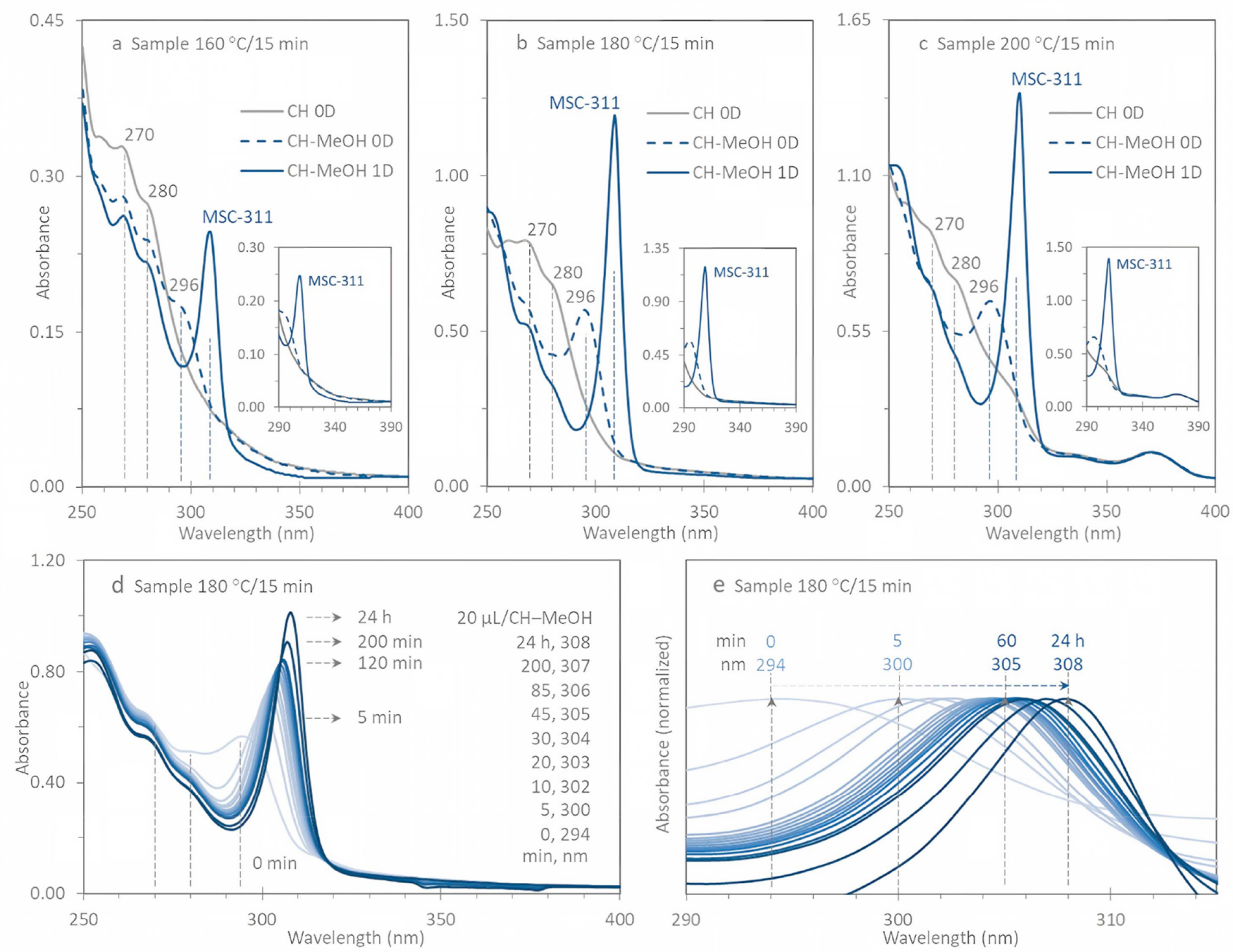
Isomerization from the CdS PNC to MSC-311 via MSC-293 in dispersion at room temperature. The three samples in the top panels (a–c) were from the Figure S5-3 reaction batch. The sample in the bottom panels (d) and (e) was from another batch kept at 180 °C. For the measurement of optical absorption (a–c), an aliquot (20 μL) of each sample was placed in CH (0D gray traces) as well as in CH–MeOH (0D dashed traces and 1D blue traces). Each inset highlights the conventional measurement from 290 nm to longer wavelengths. For the measurement of *in situ* time-resolved spectra of optical absorption (d), the sample (20 μL) was placed in CH–MeOH. The spectra were collected every 5 min from 0 to 120 min, and at 200 min and 24 h two spectra were collected. Panel (d) has 15 of the 27 spectra, and the continuous redshift from 294 to 308 is highlighted in (e) (normalized), with the spectra collected at 0, 5, and 60 min indicated, together with that at 24 h. The decrease of the absorption strength between 260 and 290 nm was accompanied by the development of MSC-293 and MSC-311.

In panels a–c of Figure 6, each panel has three spectra for Samples 160 °C (a), 180 °C (b), and 200 °C (c). The gray traces were from CH (0D), dashed traces from CH–MeOH (0D), and blue traces from CH–MeOH (1D). In CH–MeOH, the MSCs seen were MSC-293 (a–c, dashed traces) and MSC-311 (a–c, blue traces); within 1 day (blue traces), MSC-293 disappeared and MSC-311 evolved. We suggest that the PNC isomerized to MSC-293 first (dashed traces) (Scheme 1b Steps 1/2). For the appearance of MSC-311 (a–c, blue traces), there were two pathways that proceeded together. One pathway was the direct isomerization from MSC-293 to MSC-311 (Scheme 1b Step 5) (panels d and e of Figure 6). The other pathway was Scheme 1b Steps 1/3/4.

When a sample was dispersed (Figure 6a–c), the absorption strength between 260 and 290 nm was greater in CH (gray traces) than in CH–MeOH (dashed traces). This should be related to the PNC to PC/MSC-293 isomerization. Along with the evolution of the MSC-311 in CH–MeOH (blue traces), the absorption between 260 and 295 nm decreased (from the dashed to blue traces). From Sample 160 °C (a) to Sample 200 °C (c), the amount of MSC-311 increased (blue traces). This agrees with the increase of the PNC amount from Sample 160 °C to Sample 200 °C, as indicated by the strength enhancement between 260 and 290 nm (gray traces from a to c). The more the PNC forms, the more the MSC develops. During the PNC–PC/MSC transformation in dispersion, the absorption strength between 260 and 290 nm decreased. In a side note, the amount of MSC-311 developed is larger in CH–MeOH than that in CH (Figure S6-1) after 1 day.

In panels d and e of Figure 6, Sample 180 °C/15 min was studied, which was from a different batch of the Figure 5 reaction kept at 180 °C for 15 min. For the measurement of *in situ* time-resolved spectra of optical absorption, an aliquot (20 μL) of the sample was placed in CH–MeOH. Twenty-five spectra were collected every 5 min from 0 to 120 min; at 200 min and 24 h, two spectra were measured. Among the 27 spectra (Figure S6-3), 15 spectra are presented in Figure 6d, with attention to the continuous redshift from 294 to 308 nm (Figure 6e, normalized). At 0 min, MSC-293 evolved (Scheme 1b Steps 1/2) and transformed to MSC-311 directly (Scheme 1b Step 5). Meanwhile, MSC-311 developed from PC-293 to MSC-311 via PC-311 (Scheme 1b Steps 3/4).

It is of help to point out that the PNC from the two reactions, Cd­(OA)_2_ + C_12_H_25_–SH (Figures 1–4) and Cd­(OA)_2_ + S (Figures 5 and 6), isomerized to MSC-293 and MSC-311. As shown in Table S2, MSC-311, MSC-322, MSC-345, and MSC-360 were seen with various reactions, including Cd­(MA)_2_ (cadmium myristate) + S in ODE;^44^ these MSCs are isomers. We conclude that the molecule–PNC–MSC transition was followed; the core composition of the PNC was similar. Experimental parameters such as ligands and precursors of Cd and S do not change the formation pathway of the PNC and the composition of the PNC.

Classical nucleation theories (CNTs) were developed originally for vapor–liquid condensation,^5^ without bond-breaking and bond-forming events addressed. For the development of QDs and MSCs from mixtures of individual cation and anion containing molecules, chemical reactions occur. In the explanation of the N/G of QDs and the appearance of MSCs, the mainstream idea (based on the one-step LaMer model) favors the sequence of molecules → monomers → QDs and MSCs.^5–7,12^ The two-step model promotes the sequence of molecules → PNCs → monomers → QDs and PNCs → MSCs (Scheme 1a).^28–31^ The key difference between the mainstream idea and our concept is monomer formation. Our spectroscopic evidence of the CdS PNC supports the two-step model (Scheme 1a).^28–31^ We are actively exploring the spectroscopic evidence of the PNC of other II–VI semiconductors. The knowledge of the formation and transformation of the PNC in reactions to QDs would assist the development of mechanism-guided design and the advance of the synthesis from an empirical art to a science. This idea is depicted in Scheme 2. It is challenging or even technically impossible to characterize reaction intermediates, such as carbonium ions and carbanions in organic and polymer reactions, as well as the PNC in QD reactions.

**Scheme 2:**
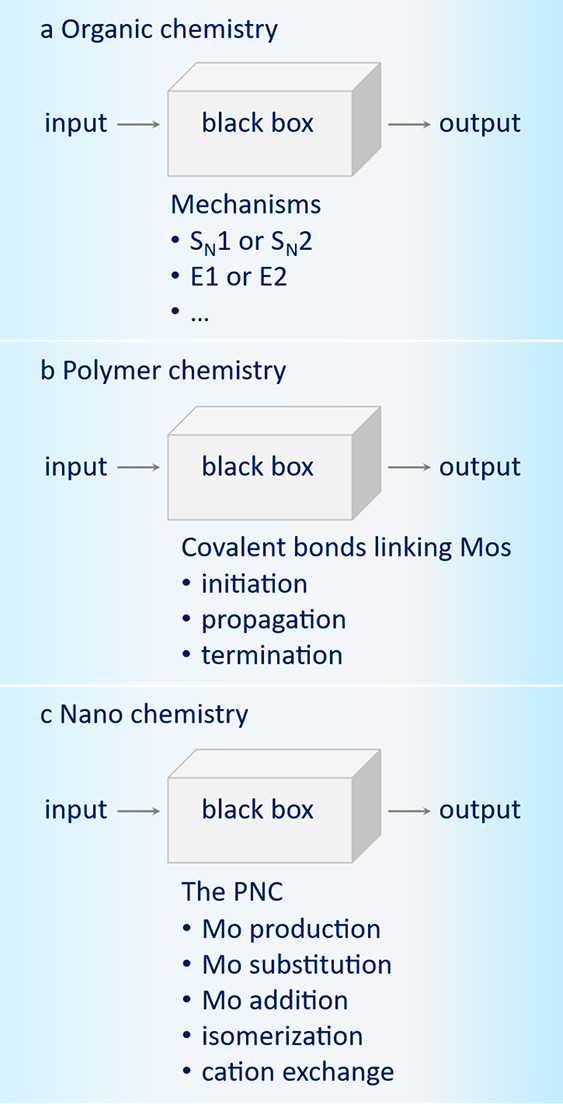
The Advance from an Empirical Art to a Science due to the Establishment of Important Concepts for (a) Organic Chemistry,^a^ (b) Polymer Chemistry,^b^ and (c) Nanochemistry^c^

a For organic chemistry,^71,72^ the concepts include substitution nucleophilic unimolecular reactions (S_N_1), substitution nucleophilic bimolecular reactions (S_N_2), unimolecular elimination reactions (E1), and bimolecular elimination reactions (E2). Physical organic chemistry developed.
b For polymer chemistry,^73,74^ the concepts include how monomers are linked by covalent bonds, together with the process of initiation, propagation, and termination.
c For nanochemistry,^28–31,75,76^ the concepts include chemical self-assembly resulting in the stable PNC. Furthermore, the PNC may become monomers and/or experience monomer substitution, monomer addition, isomerization, and cation exchange.

## Conclusion

Using optical absorption spectroscopy, we have provided the first spectroscopic evidence that compellingly supports the formation and transformation of the PNC. The appearance of the PNC was studied with two model reactions of Cd­(OA)_2_ + CH_3_(CH_2_)_11_–SH (Figure 1) and Cd­(OA)_2_ + S (Figure 5). When the temperature of the two reactions was above 200 and 160 °C, respectively, the PNC formed (Scheme 1a Steps 1a/1b) with the enhanced absorption between 260 and 290 nm. When a sample (with the PNC) was placed in CH–MeOH (Figures 2 and 6), the PNC transformed to MSC-293 (Scheme 1b Steps 1/2) and MSC-311 (Scheme 1b Steps 1/3/4). With an isosbestic point at ∼288 nm, the absorption strength between 260 and 290 nm decreased due to the consumption of the PNC. MSC-293 might transform to MSC-311 directly (Scheme 1b Step 5), featuring an uninterrupted redshift. When MSC-293 transformed to MSC-311 indirectly (Scheme 1b Steps 2′/3/4), a sporadic redshift was seen (Figure 3). When MSC-311 transformed to MSC-293 indirectly (Scheme 1b Steps 4′/3′/2), a stepwise blueshift was seen (Figure 4). With little change of the absorption strength between 260 and 290 nm, these two PC-assisted transformations had an isosbestic point at ∼300 nm.

The present findings in conjunction with our previous work^28–31,41–58^ lay a solid foundation for the appearance of the PNC that is uniquely driven by chemical self-assembly in reactions of colloidal semiconductor QDs (Scheme 1a). It is our conviction that this study will ultimately enable a transformative capability to better understand chemical self-assembly,^4,32–36^ which leads to the molecule–PNC transition in reactions of colloidal semiconductor QDs, the prenucleation stage of which was depicted as a “forbidden region” and a “black box” in the past,^6,43^ as shown by Scheme 2c. The knowledge gained about the molecule–PNC–solid transition will motivate, engage, and catalyze the enthusiastic exploration of chemical self-assembly^4,32–36^ in the colloidal synthesis of functional nanomaterials with high performance that steers in the improvement of the existing applications and the empowerment of emergent ones.^67–70^

## Supplementary Material



## Notes

Experimental details of synthesis and characterization, including optical absorption; literature summary of CdS MSCs; and additional data, including absorbance spectra and numerical analysis (PDF)
