# Supplementary material for: Spectroscopic Evidence in Optical Absorption for the Formation and Transformation of Prenucleation Clusters of CdS Quantum Dots
Source: ACS Cent Sci. 2026 Jun 29;12(7):996–1007. doi: 10.1021/acscentsci.6c00394 (PMC13397445; doi:10.1021/acscentsci.6c00394)
Supplement: Supplementary file 1 [file oc6c00394_si_001.pdf]

Supporting Information

Spectroscopic Evidence in Optical Absorption for the Formation and Transformation of Prenucleation Clusters of CdS Quantum Dots

Yang Li,[1] Kui Yu,[*,1] Yusha Yang,[1] Andrei Sapelkin,[2]
Sanwei Guo,[3] Xiaobo Zhu,[3] Sijie Zhang,[4] Xiaoqin Chen[*,1]

[1]Engineering Research Center in Biomaterials, Sichuan University,
Chengdu, Sichuan, 610065, P. R. China
[2]Department of Physics and Astronomy, Queen Mary, University of London
327 Mile End Road, London, E1 4NS, United Kingdom
[3]Cannano Jiayuan (Guangzhou) Science & Technology Co. Ltd,
Guangzhou 510700, P. R. China
[4]Department of Physics, Guizhou University of Engineering Science
Bijie, Guizhou, 551700, P. R. China

*To whom correspondence should be addressed to
K. Y. (email: kuiyu@scu.edu.cn) and X. C. (email: xqchen@scu.edu.cn)

Table of Contents

**Experimental Section**		S02
**Table S1**	Summary of the acronyms	S04
**Table S2**	CdS MSC-293 not reported previously	S06
**Figure SA**	Absorption spectra of a 2025 Angew study on CdS (Ref 62)	S10
**Figure SB**	Absorption spectra of a 2002 Angew study on CdS (Ref 61)	S11
**Figure S1-1**	The N/G of QDs above 260 °C for the Figure 1a reaction	S12
**Figure S1-2A**	Absorption comparison for Samples 60 to 160 °C	S13
**Figure S1-2B**	Absorption of samples heated at 220 °C	S14
**Figure S1-3A**	Spectra after the background subtraction	S15
**Figure S1-3B**	The numerical analysis supporting the PNC formation	S16
**Figure S1-4**	Four full sets of spectra of the Figure 1a reaction sample	S17
**Figure S2-1**	Absorption comparison of the Figure 2 a–c sample in CH	S19
**Figure S2-2**	Absorption comparison for Samples 160 to 200 °C	S20
**Figure S2-3**	The full set of spectra for Parts d and e of Figure 2	S21
**Figure S3-1**	Isomerization of the PNC (220 °C/60 min) in dispersion	S22
**Figure S3-2**	Isomerization of the PNC (220 °C/120 and 150 min) in dispersion	S23
**Figure S4-1**	MSC-293 to MSC-311 isomerization	S24
**Figure S4-2**	The Figure 4b sample in dispersion after 5-day storage	S25
**Figure S5-1**	Additional information for samples from the Figure 5a reaction	S26
**Figure S5-2**	Spectra after the background subtraction	S27
**Figure S5-3**	Four full sets of spectra for another Figure 5a batch samples	S28
**Figure S6-1**	Absorption comparison of the Figure 6 a–c sample in CH	S29
**Figure S6-2**	Absorption comparison for Figure S5-3 Samples 100 to 140 °C	S30
**Figure S6-3**	The full set of spectra for Parts d and e of Figure 6	S31

Experimental Section

Chemicals. Cadmium oxide (CdO, 99.99%), oleic acid (OA, 90%), and 1-octadecene (1-ODE, 90%) were purchased from Sigma-Aldrich. 1-Dodecanethiol ($C_{12}H_{25}$−SH, 98%) was from Adamas-beta. Sulfur powder (S, 99.5%), methanol (MeOH, 99.5%), and cyclohexane (CH, 99.5%), were obtained from Chengdu Ke Long Chemical. All chemicals were used as received without purification.

$Cd(OA)_2$ stock solution preparation. CdO (1.5413 g, 12.00 mmol), OA (7.459 g, 26.41 mmol), and ODE (10.019 g) were loaded in a 100 mL three-necked flask equipped with a condenser. The resulting mixture was stirred at room temperature. The flask was evacuated for ~8 min until no bubbles were observed. The flask was backfilled with nitrogen (N_2) for 2 min. This procedure was repeated three times within 30 min. Under a N_2 atmosphere, the mixture was heated to ~75 °C, and was then evacuated for one hour. Under a N_2 atmosphere, the mixture was heated to 120 °C, and was then evacuated for one hour. The mixture was then further heated to 240 °C under N_2 and kept for 40 min. The resulting solution was transparent with a light-yellow color, and had a concentration of $Cd(OA)_2$ of 0.631 $mmol\cdot g^{-1}$. Under a N_2 atmosphere, the solution was cooled to room temperature and stored in one refrigerator at 4 °C for future usage. The use of the stock solution ensured that similar $Cd(OA)_2$ was used such as for the study shown in Figure 1 and for the study shown in Figure 5. Three reaction batches of $Cd(OA)_2$ + $C_{12}H_{25}$−SH (b−f burgundy traces), $Cd(OA)_2$ (b−f blue traces), and $C_{12}H_{25}$−SH (b−f grey traces) were dealt with for Figure 1. Three reaction batches of $Cd(OA)_2$ + S (b−f burgundy traces), $Cd(OA)_2$ (b−f blue traces), and S (b−f burgundy traces) were dealt with for Figure 5. Experimental conditions such as the vacuum power affect the reaction CdO + HOA $\rightarrow$ $Cd(OA)_2$ + H_2O.

Synthesis of CdS samples. For the Figure 1a synthesis, a mixture of $Cd(OA)_2$ (0.952 g, 0.60 mmol) and ODE (4.043 g) was placed in a 50 mL three-necked flask equipped with a condenser. At room temperature, the flask was evacuated for ~8 min (until no bubbles were seen) and was then backfilled with nitrogen (N_2) for 2 min. This procedure was repeated three times within 30 min. Under a N_2 atmosphere, the mixture was stirred and heated to 120 °C and was evacuated for one hour (until no bubbles seen). Under a N_2 atmosphere, the

$Cd(OA)_2$ solution was cooled down to 30 °C. $C_{12}H_{25}$–SH (36 μL, 0.15 mmol) was added into the mixture. The resultant mixture had a total weight of 5.006 g, with a feed molar ratio of 4Cd−1S and a feed S concentration of 30 mmol·Kg^{-1}. The reaction temperature increased from 40 to 260 °C with a step of 20 °C; samples were extracted after 15 min at each step. Another batch was performed with the reaction temperature increased to 240 °C and kept for 15 min and then to 260 °C for 15 min (Parts d and e of Figure 2). Another batch was performed with the reaction temperature increased to 220 °C; sampling was after 60 min (Figure S3-1), 90 min (Figure 3), 120 min (Part a of Figure S3-2), and 150 min (Part b of Figure S3-2). Another batch was performed with the reaction temperature increased to 220 °C; sampling was after 120 min (Part a of Figure 4). Another batch was performed with similar sampling to that of the Figure 1a batch (Part b of Figure 4).

For the Figure 5a synthesis, a mixture of $Cd(OA)_2$ (0.962 g, 0.61 mmol) and ODE (4.039 g) was placed in a 50 mL three-necked flask equipped with a condenser. At room temperature, the flask was evacuated for ~8 min (until no bubbles seen) and was then backfilled with nitrogen (N_2) for 2 min. This procedure was repeated three times within 30 min. Under a N_2 atmosphere, the mixture was stirred and heated to 120 °C and was evacuated for one h (until no bubbles seen). Under a N_2 atmosphere, the $Cd(OA)_2$ solution was cooled down to 80 °C, and S (0.0052 g, 0.16 mmol) was added. The resultant mixture had a total weight of 5.0062 g, with a feed molar ratio of 4Cd−1S and a feed S concentration of 32 mmol·Kg^{-1}. The reaction temperature increased from 80 to 220 °C with a step of 20 °C; samples were extracted after 15 min at each step (Figure 5). Another batch was performed (Parts a to c of Figure 6). Also, another batch was performed with the reaction temperature directly to 180 °C and kept for 15 min (Parts d and e of Figure 6).

Ultraviolet-visible (UV-Vis) absorption spectroscopy. UV-vis absorption spectra were collected between 250 and 500 nm with an interval of 1 nm. Hitachi UH4150 and Hitachi U-2910 spectrometers were used. The quartz cuvettes (3.5 mL standard QS cells with the light path of 10 mm) were purchased from Hellma Analytics. Usually, an aliquot of a sample was dispersed in 3.0 mL of CH (3.0CH) or a mixture of 3.0 mL CH and 10 μL MeOH (CH–MeOH). Baseline measurements were performed with CH.

Table S1. Additional explanations for the PNC, PC, MSC, and monomer (Mo).

Acronym (full name)	LaMer Model	Yu Model	Nature
PNC (prenucleation cluster)	NA	relatively disordered, forming via chemical self-assembly; can be the PC of one type of MSCs	transparent at the peak position of the corresponding MSC absorption and to longer wavelengths
PC (precursor compound)	NA	relatively disordered, a MSC counterpart	transparent at the peak position of the corresponding MSC absorption and to longer wavelengths
MSC (magic-size cluster)	NA	relatively ordered, evolving from a relatively disordered state (PC)	displaying sharp optical absorption at persistent positions
Mo (monomer)	M_1E_1, forming via the direct reaction of M and E precursors[1-5]	from the PNC;[6-11] M_2E_n, n = 1 (Cu), 2 (Cd, Zn, Pb, Ge), and 3 (In)[5]	with ligands, but the composition unknow

The PNCs, PCs, MSCs, and monomers of CdS have Cd–S bonds. CdS MSC-293 and MSC-311 have different feature of optical absorption, together with PC-293 and PC-311.

(1) Steckel, J. S.; Yen, B. K. H.; Oertel, D. C.; Bawendi, M. G. On the Mechanism of Lead Chalcogenide Nanocrystal Formation. *J. Am. Chem. Soc.* **2006**, *128*, 13032–13033.

(2) Reiss, P. ZnSe Based Colloidal Nanocrystals: Synthesis, Shape Control, Core/Shell, Alloy and Doped Systems. *New J. Chem*. **2007**, *31*, 1843–1852.

(3) Owen, J. S.; Chan, E. M.; Liu, H.; Alivisatos, A. P. Precursor Conversion Kinetics and the Nucleation of Cadmium Selenide Nanocrystals. *J. Am. Chem. Soc.* **2010**, *132*, 18206–18213.

(4) García-Rodríguez, R.; Hendricks, M. P.; Cossairt, B. M.; Liu, H.; Owen, J. S. Conversion Reactions of Cadmium Chalcogenide Nanocrystal Precursors. *Chem. Mater.* **2013**, *25*, 1233–1249.

(5) Yu, K.; Liu, X.; Qi, T.; Yang, H.; Whitfield, D. M.; Chen, Q. Y.; Huisman, E. J.C.; Hu, C. General Low-Temperature Reaction Pathway from Precursors to Monomers before Nucleation of Compound Semiconductor Nanocrystals. *Nat. Commun.* **2016**, *7*, 12223.

(6) Zhang, J.; Hao, X.; Rowell, N.; Kreouzis, T.; Han, S.; Fan, H.; Zhang, C.; Hu, C.; Zhang, M.; Yu, K. Individual Pathways in the Formation of Magic-Size Clusters and Conventional Quantum Dots. *J. Phys. Chem. Lett*. **2018**, *9*, 3660–3666.

(7) Sun, X.; Wang, S.; Wang, Z.; Shen, Q.; Chen, X.; Chen, Z.; Luan, C.; Yu, K. Lower-Temperature Nucleation and Growth of Colloidal CdTe Quantum Dots Enabled by Prenucleation Clusters with Cd–Te Bond Conservation. *J. Am. Chem. Soc*. **2024**, *146*, 15587–15595.

(8) Shen, Q.; Yu, K.; Liu, Y.; Chen, Z.; Sapelkin, A.; Luan, C.; Chen, X. Nucleation and Growth

of ZnSe Quantum Dots from Prenucleation Clusters in Dispersion at Room Temperature. *Adv. Funct. Mater.* **2025**, *35*, 2504115.

(9) Wang, M.; Yu, K.; Sapelkin, A.; Shen, Q.; Liang, B.; Yuan, S. Prenucleation Cluster Effects on Colloidal CdSe Semiconductor Samples in Dispersion at Room Temperature. *Small* **2025**, *21*, 2503510.

(10) Feng, W.; Yu, K.; Li, Y.; Sapelkin, A.; Zhu, X.; Chen, X. Similar Prenucleation Clusters in Hot-Injection and Heating-Up Approaches to CdS Colloidal Semiconductor Quantum Dots. *J. Phys. Chem. Lett.* **2026**, *17*, 5346–5354.

(11) Liu, Y.; Yu, K.; Yang, Y.; Sapelkin, A.; Guo, S.; Zhang, S.; Chen, X. Environmental Polarity Regulating Development of Magic-Size Clusters and Quantum Dots from CdTe Prenucleation Clusters. *Inorg. Chem.* **2026**. *65*, 9145–9154.

Table S2. CdS MSC-293 not reported previously. Literature summary of the CdS MSCs reported from reactions at elevated temperatures and from dispersion at room temperature (RT).[1-14]

Refs year	MSCs Cd to S ratios	Reactions (°C)	Comments
**1** 2025	322: $Cd_{28}S_{17}$ chiral MSCs	$CdI_2(PEt_3)_2$ + [S–Cu_{50}] (RT)	enantiomerically biased crystallization single-crystal XRD
**2** 2024	322: $Cd_{41}S_{20}$ 345: NA 360: $Cd_{41}S_{20}$	$Cd(MA)_2$, S powder (140, 180)	isomers, MALDI, EDX 322 at 180 °C 345 at RT, 360 at RT
**3** 2023	322: Cd:S = 2.2	$Cd(MA)_2$, S powder (160)	ICP-OES 160 °C
**4** 2022	311: $Cd_{37}S_{32}$	ZnS sample + $Cd(OA)_2$ (RT)	MALDI–TOF–MS
**5** 2018	311: Cd:S = 2.4 322: Cd:S = 2.5	$Cd(OA)_2$, S powder (180)	isomers MALDI, ICP–OES, 311 at 4 °C, 322 at 60 °C
**6** 2022	311: $Cd_{41}S_{20}$ 322: $Cd_{41}S_{20}$	NA	Following Ref 7 DFT computation
**7** 2017	311: Cd:S = 1.9 322: Cd:S = 2.0 348: Cd:S = 2.0 360: Cd:S = 1.2	$Cd(OA)_2$, STOP (322 at 140)	ICP–OES 322 + C_2H_5OH → 311 322 + $C_{12}H_{25}SH$ → 348 322 + $C_4H_9NH_2$ → 360
**8** 2018	322: $Cd_{17\text{-}32}S$ Cd:S = 2.0	$Cd(OA)_2$, STOP (130)	17 to 32 Cd atoms per cluster
**9** 2019	311: $Cd_{37}S_{20}$ 322: $Cd_{37}S_{20}$	$Cd(OA)_2$, STOP (140)	Based on $In_{37}P_{20}$ MSCs[14]
**10** 2022	311: $Cd_{37}S_{18}$ 322: $Cd_{37}S_{18}$	$Cd(OA)_2$, STOP (130)	No explanation for $Cd_{37}S_{18}$
**11** 2023	311: $Cd_{13}S_{13}$	$Cd(OAc)_2/RNH_2$ S-OTA (RT)	LDI–TOF–MS ICP–OES
**12** 2022	335: $Cd_{34}S_{34}$ 365: $Cd_{34}S_{34}$	$CdCl_2$/BTA, S-BTA (40)	LDI–TOF–MS ICP–OES
**13** 2015	361: $Cd_{34}S_{34}$	$Cd(OAc)_2/RNH_2$ TAA (60)	LDI–TOF–MS

[S–Cu_{50}]: ($Cu_{50}S_{12}(SC(CH_3)_3)_{20}(CF_3COO)_{12}$)
PEt_3: triethylphosphine
$Cd(MA)_2$: cadmium myristate
$Cd(OA)_2$: cadmium oleate
TAA: thioacetamide

The Cd to S number ratio and composition of these reported CdS MSCs remain elusive, together with their structures. It is challenging to have intact clusters after purification. Their surface ligands are labile and their cores are dynamic.[15] In the case of the PNC, it may change during purification via the transformation of PNCs → MSCs and/or PNCs →

monomers → QDs; it is impossible to keep the PNC intact after purification. Furthermore, $Cd(OA)_2$ in ODE can form a supramolecular gel,[16] the removal of which is difficult during purification of either QDs or clusters. Therefore, it is technically impossible to have conclusive information of the composition and structure of a cluster (including the PNC and the MSC).

For CdS MSC-311,[4-7,9-11,17-19] its composition and structure remains elusive after exhaustive characterization including matrix-assisted laser desorption/ionization time-of-flight (MALDI-TOF) mass spectrometry (MS), Cd K-edge X-ray absorption fine structure (XAFS), energy dispersive X-ray spectroscopy (EDX), thermogravimetric analysis (TGA), inductively coupled plasma optical emission spectroscopy (ICP-OES), X-ray total scattering with atomic pair distribution function (PDF) analysis, transmission electron microscopy (TEM), powder X-ray diffraction (XRD), small angle X-ray scattering (SAXS), powder wide angle X-ray scattering (WAXS), and in situ optical absorption spectroscopy.

MSC-311, MSC-322, MSC-345, and MSC-360 are isomers.[1,5,11] Thus, experimental parameters, such as reaction temperatures, media, as well as individual Cd-containing and S-containing molecules at lease used so far, do not change the formation and the composition of the CdS PNC (although unknown at present). This idea is supported by a recent study.[20]

This view is general. Because from different reactions of ZnSe, the PNC isomerized to ZnSe MSC-299.[21-23] From different reactions of CdSe, the PNC isomerized to CdSe MSC-330, MSC-360, MSC-390, and MSC-415.[24-26]

(1) Xu, C.; Zhang, Z.; Zhou, Z.; Han, H. A Chiral CdS Magic-Size Cluster with Enantiomerically-Biased Crystallization. *J. Am. Chem. Soc.* **2025**, *147*, 17890–17901.

(2) Xu, R.; Wang, Z.; Yang, Y.; Gu, C.; Luan, C.; Wang, S.; Chen, X.; Yu, K. Formation and Transformation of CdS Clusters during the Prenucleation Stage and in a Dilute Dispersion at Room Temperature. *Nano Lett.* **2024**, *24*, 1294–1302.

(3) Wang, D.; Liu, Y.; Rowell, N.; Wang, S.; Zhang, C.; Zhang, M.; Luan, C.; Yu, K. Direct and Indirect Evolution of Photoluminescent Semiconductor CdS Magic-Size Clusters through Their Precursor Compounds. *Angew. Chem. Int. Ed.* **2023**, e202304329.

(4) He, L.; Luan, C.; Liu, S.; Chen, M.; Rowell, N.; Wang, Z.; Li, Y.; Zhang, C.; Lu, J.; Zhang, M.; Liang, B.; Yu, K. Transformations of Magic-Size Clusters via Precursor Compound Cation Exchange at Room Temperature. *J. Am. Chem. Soc.* **2022**, *144*,19060–19069

(5) Zhang, B.; Zhu, T.; Ou, M.; Rowell, N.; Fan, H.; Han, J.; Tan, L.; Dove, M. T.; Ren, Y.; Zuo, X.; Han, S.; Zeng, J.; Yu, K. Thermally-Induced Reversible Structural Isomerization in Colloidal Semiconductor CdS Magic-Size Clusters. *Nat. Commun.* **2018**, *9*, 2499.

(6) Shim, D.; Lee, J.; Kang, J. Multiscale Isomerization of Magic-Sized Inorganic Clusters Chemically Driven by Atomic-Bond Exchanges. *Chem. Mater.* **2022**, *34*, 9527–9535.

(7) Nevers, D. R.; Williamson, C. B.; Hanrath, T.; Robinson, R. D. Surface Chemistry of Cadmium Sulfide Magic-Sized Clusters: A Window into Ligand-Nanoparticle Interactions. *Chem. Commun.* **2017**, *53*, 2866–2869.

(8) Nevers, D. R.; Williamson, C. B.; Savitzky, B. H.; Hadar, I.; Banin, U.; Kourkoutis, L. F.;

Hanrath, T.; Robinson, R. D. Mesophase Formation Stabilizes High-Purity Magic-Sized Clusters. *J. Am. Chem. Soc.* **2018**, *140*, 3652–3662.
(9) Williamson, C. B.; Nevers, D. R.; Nelson, A.; Hadar, I.; Banin, U.; Hanrath, T.; Robinson, R. D. Chemically Reversible Isomerization of Inorganic Clusters. *Science* **2019**, *363*, 731–735.
(10) Han, H.; Kallakuri, S.; Yao, Y.; Williamson, C. B.; Nevers, D. R.; Savitzky, B. H.; Skye, R. S.; Xu, M.; Voznyy, O.; Dshemuchadse, J.; Kourkoutis, L. F.; Weinstein, S. J.; Hanrath, T.; Robinson, R. D. Multiscale Hierarchical Structures from a Nanocluster Mesophase. *Nat. Mater.* **2022**, *21*, 518–525.
(11) Deng, Y.; Liang, J.; Kong, X.; Xiao, P.; Zhou, Y.; Wang, Y. Unraveling the Transformation Pathways in Semiconductor Clusters by Studying the Formation of Spectroscopically Pure $(CdS)_{13}$ Magic-Size Clusters. *Chem. Mater.* **2023**, *35*, 2463–2471.
(12) Ge, J.; Liang, J.; Chen, X.; Deng, Y.; Xiao, P.; Zhu, J.; Wang, Y. Designing Inorganically Functionalized Magic-Size II–VI Clusters and Unraveling Their Surface States. *Chem. Sci.* **2022**, *13*, 11755–11763.
(13) Wang, Y.; Zhou, Y.; Zhang, Y.; Buhro, W. E. Magic-Size II-VI Nanoclusters as Synthons for Flat Colloidal Nanocrystals. *Inorg. Chem*. **2015**, *54*, 1165–1177.
(14) Gary, D. C.; Flowers, S. E.; Kaminsky, W.; Petrone, A.; Li, X.; Cossairt, B. M. Single-Crystal and Electronic Structure of a 1.3 nm Indium Phosphide Nanocluster. *J. Am. Chem. Soc.* **2016**, *138*, 1510–1513.
(15) Mech, S. A.; Gibson, J. O.; Ma, F.; Dobrzycki, L. M.; Zeng, C. From Magic Size to Atomic Precision: Facile Synthesis of a CdTe Semiconductor Nanocluster. *J. Am. Chem. Soc.* **2025**, *147*, 7507–7512.
(16) Welsch, T. A.; Cleveland, J. M.; Thomas, J. A.; Schyns, Z. O. G.; Korley, L. T. J.; Doty, M. F. Supramolecular Gelation of Cadmium Oleate in the Synthesis of Nanocrystals for Applications in Photonics and Optoelectronics. *ACS Appl. Nano Mater*. **2024**, *7*, 13319–13327.
(17) Zhu, T.; Zhang, B.; Zhang, J.; Lu, J.; Fan, H.; Rowell, N.; Ripmeester, J. A.; Han, S.; Yu, K. Two-Step Nucleation of CdS Magic-Size Nanocluster MSC-311. *Chem. Mater.* **2017**, *29*, 5727–5735.
(18) Zhang, J.; Hao, X.; Rowell, N.; Kreouzis, T.; Han, S.; Fan, H.; Zhang, C.; Hu, C.; Zhang, M.; Yu, K. Individual Pathways in the Formation of Magic-Size Clusters and Conventional Quantum Dots. *J. Phys. Chem. Lett.* **2018**, *9*, 3660−3666.
(19) Lynch, R. P.; Ugras, T. J.; Robinson, R. D. Discovery of Isomerization Intermediates in CdS Magic-Size Clusters. *ACS Nano* **2024**, *18*, 27524–27536.
(20) Feng, W.; Yu, K.; Li, Y.; Sapelkin, A.; Zhu, X.; Chen, X. Similar Prenucleation Clusters in Hot-Injection and Heating-Up Approaches to CdS Colloidal Semiconductor Quantum Dots. *J. Phys. Chem. Lett.* **2026**, *17*, 5346–5354.
(21) Shen, Q.; Yu, K.; Liu, Y.; Chen, Z.; Sapelkin, A.; Luan, C.; Chen, X. Nucleation and Growth of ZnSe Quantum Dots from Prenucleation Clusters in Dispersion at Room Temperature. *Adv. Funct. Mater.* **2025**, *35*, 2504115.
(22) Zhao, D.; Wang, S.; Xue, J.; Zhang, C.; Wang, S.; Chen, X.; Luan, C.; Yu, K. Formation of ZnSe Magic-Size Clusters Displaying Optical Absorption Doublets from Prenucleation Clusters. *Nano Res.* **2024**, *17*, 6741–6748.
(23) Wang, S.; Wang, Z.; Xue, J.; Chen, X.; Luan, C.; Yu, K. A Prenucleation-Stage Sample of ZnSe Assisting Lower-Temperature Shell Growth on CdSe Magic-Size Clusters via Monomer Addition. *Small* **2024**, *21*, 2408285.

(24) Zhu, D.; Hui, J.; Rowell, N.; Liu, Y.; Chen, Q. Y.; Steegemans, T.; Fan, H.; Zhang, M.; Yu, K. Interpreting the Ultraviolet Absorption in the Spectrum of 415 nm-Bandgap CdSe Magic-Size Clusters. *J. Phys. Chem. Lett.* **2018**, *9*, 2818–2824.

(25) Zhang, L.; Yu, K.; Yang, Y.; Sapelkin, A. V.; Wang, Z.; Luan, C.; Chen, X. Development of CdSe Magic-Size Clusters Displaying Optical Absorption Singlets from One Prenucleation-Stage Sample in Dispersion under Mild Conditions. *Nano Res.* **2025**, *18*, 94907837.

(26) Wang, M.; Yu, K.; Sapelkin, A.; Shen, Q.; Liang, B.; Yuan, S. Prenucleation Cluster Effects on Colloidal CdSe Semiconductor Samples in Dispersion at Room Temperature. *Small* **2025**, *21*, 2503510.

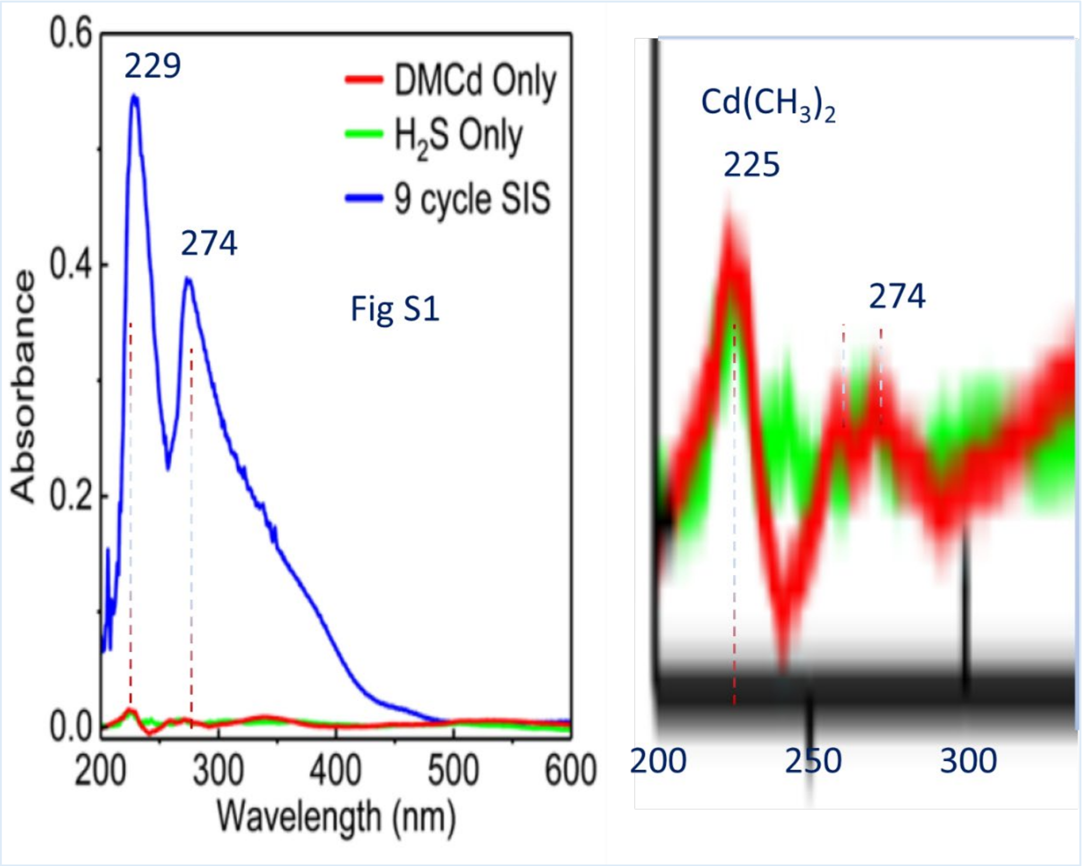

Figure SA. The spectra (edited) in a study of the reaction of dimethyl cadmium (DMCd) and hydrogen sulfide (H_2S) in the polymer of poly(4-vinylpyridine) (Ref 62).[1] The left panel is from Figure S1 but enlarged. The spectra of DMCd (red trace) and H_2S (green trace) are highlighted in the right panel. With the process of sequential infiltration synthesis (SIS), the absorption strength around 275 nm of the product (blue trace) increased. The strength increase is similar to the spectroscopic evidence of the PNC from our two reactions in ODE of $Cd(OA)_2$ + $C_{12}H_{25}$–SH and of $Cd(OA)_2$ + S. We suggest that the increase of the absorption strength around 270 nm is due to the PNC formation in the reaction of DMCd + H_2S.

(1) Jayaweera, N. P.; Havenridge, S.; Bielinski, A. R.; Kim, K.; Thompson, N. B.; Hoffman, J. M.; Wheaton, A. M.; Sarkar, P.; Pathak, R.; Elam, J. W.; Liu, C.; Mulfort, K. L.; Martinson, A. B. F. Sequential Infiltration Synthesis of Cadmium Sulfide Discrete Atom Clusters. *Angew. Chem. Int. Ed.* **2025**, *64,* e202421259.

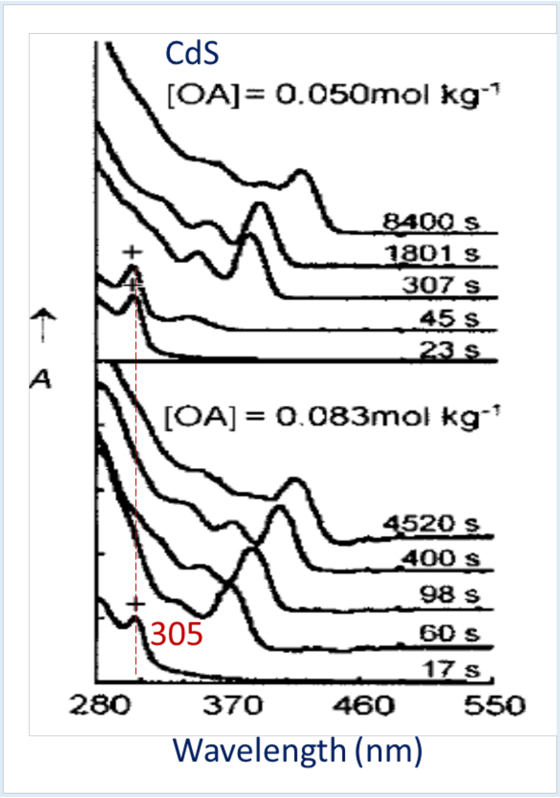

Figure SB. The spectra (edited) from Figure 1 of a study of the reaction of $Cd(OA)_2$ + S in ODE (Ref 61).[1] A = absorbance and MSCs are marked with +. A hot-injection approach was used with the injection temperature of 300 °C and the growth temperature of 250 °C. The first exciton absorption peak of the MSCs is at 305 nm. We now suggest that the marked MSCs can be the intermediate trapped when MSC-293 directly isomerizes to MSC-311.

(1) Yu, W. W.; Peng, X. Formation of High-Quality CdS and Other II–VI Semiconductor Nanocrystals in Noncoordinating Solvents: Tunable Reactivity of Monomers. *Angew. Chem. Int. Ed*. **2002,** *41*, 2368–2371.

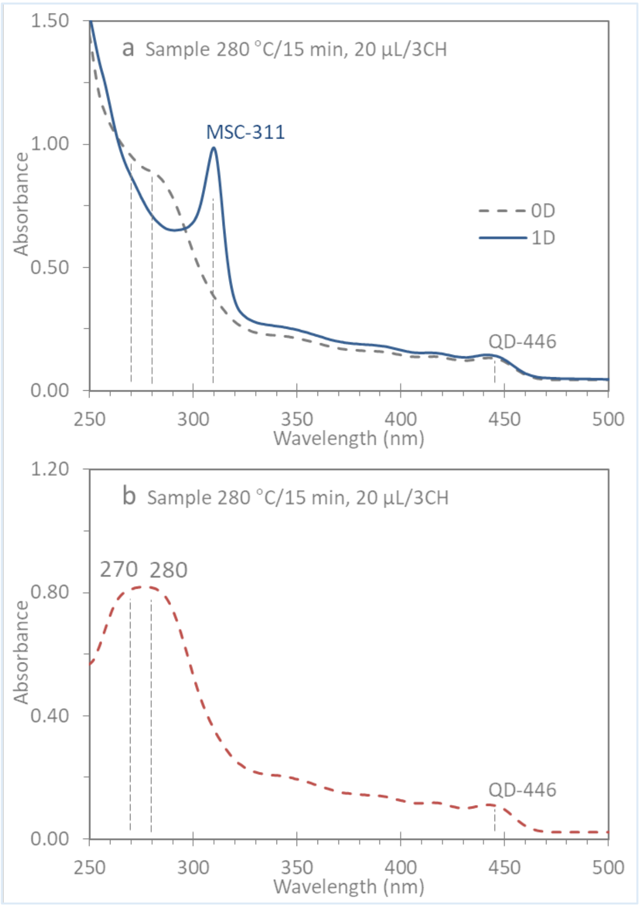

Figure S1-1. The N/G of QDs above 260 °C in the reaction of $Cd(OA)_2$ and $C_{12}H_{25}$–SH in ODE. Sample 280 °C/15 min was from a different batch of the Figure 1a reaction with similar sampling. 280 °C/15 min had QD-446 and the PNC. The PNC displays broad optical absorption around 260 and 290 nm.

- (a) The PNC isomerized to MSC-311 in CH after one day, with the decrease of the absorption strength around 260 and 290 nm (from the dashed to solid traces).
- (b) The dashed trace was obtained after the background subtraction. The trace (in CH 0D) of Sample 140 °C/15 min from this batch was deducted.
- In a side note, Sample 240 °C/15 min from this batch was used for the study shown in Figures 4b and S4-1 and S4-2.

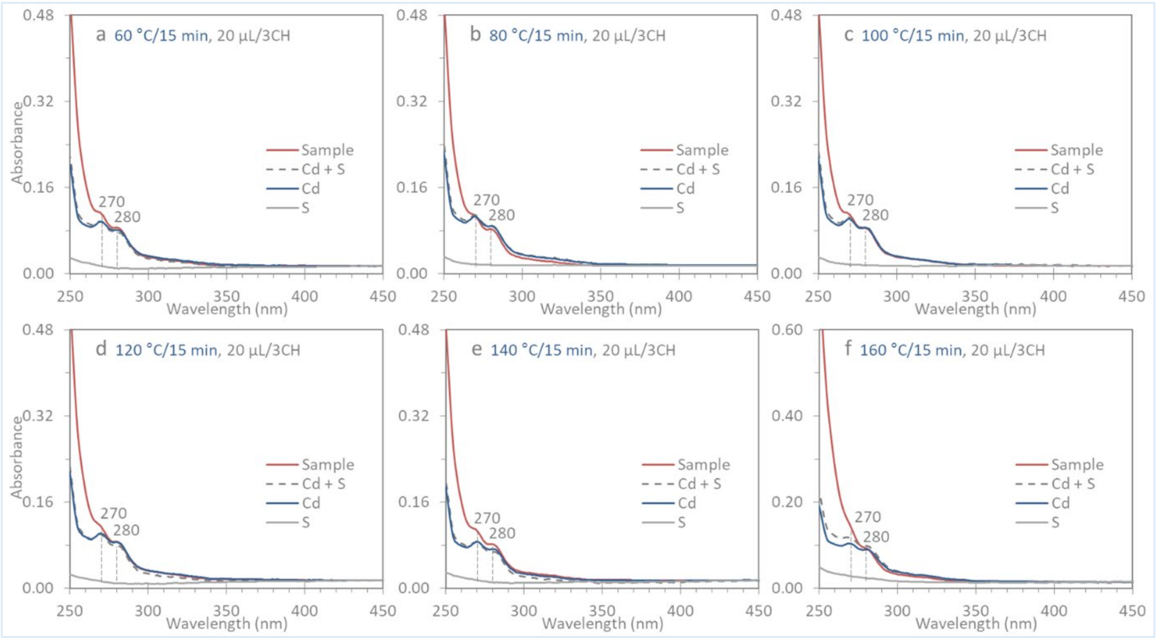

Figure S1-2A. Absorption comparison with a similar presentation format to that of Parts b–f of Figure 1. For these samples obtained at 60 to 160 °C, the difference at 270 and 280 nm is small between the sample absorption (burgundy traces) and the superimposed signal (dashed traces) of $Cd(OA)_2$ (blue traces) and $C_{12}H_{25}$−SH (grey traces). The CdS PNC formed little below 200 °C in the reaction of $Cd(OA)_2$ and $C_{12}H_{25}$−SH in ODE.

- When $Cd(OA)_2$ and $CH_3(CH_2)_{11}$−SH were mixed and heated from 60 to 160 °C, the absorption strength of the peaks at 270 and 280 nm (burgundy traces) was similar to that of $Cd(OA)_2$ without the sulfur source (blue traces) and that of the summated signal (dashed traces) of $Cd(OA)_2$ (blue traces) and $C_{12}H_{25}$−SH (grey traces). We attribute the strength similarity between the dashed traces and the burgundy traces to no formation of Cd−S bonds.
- When $Cd(OA)_2$ and $CH_3(CH_2)_{11}$−SH were mixed and heated above 200 °C (Figure 1), the absorption strength of the peaks at 270 and 280 nm (burgundy traces) became larger than that of $Cd(OA)_2$ without the sulfur source (blue traces) and that of the superimposed signal (dashed traces) of $Cd(OA)_2$ (blue traces) and $C_{12}H_{25}$−SH (grey traces). We attribute the strength increase from the dashed traces to the burgundy traces to the formation of the PNC with Cd−S bonds.
- In a side note, the optical absorption of $Cd(OA)_2$ was reported in 2002 (Ref 61), with two peaks at ~270 and 280 nm as shown on Page 3 of the supporting information file of the

2002 study.[1] The PNC has more Cd than S (Table S2). It is reasonable that with the Cd−OOR bond, the optical absorption of the PNC has the signature of $Cd(OA)_2$.

(1) Yu, W. W.; Peng, X. Formation of High-Quality CdS and Other II–VI Semiconductor Nanocrystals in Noncoordinating Solvents: Tunable Reactivity of Monomers. *Angew. Chem. Int. Ed*. **2002**, *41*, 2368–2371.

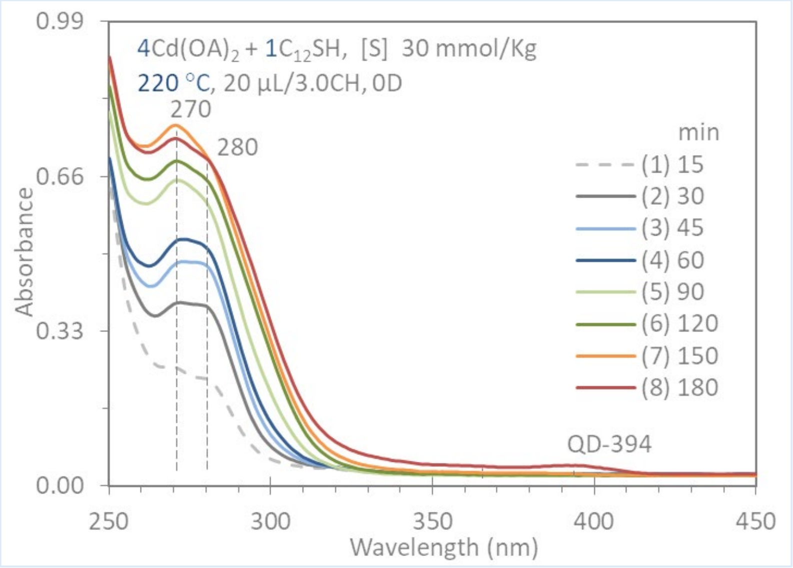

Figure S1-2B. Development of the CdS PNC at 220 °C. Figure 1 shows the development of the CdS PNC above 200 °C in the reaction of $Cd(OA)_2$ and $C_{12}H_{25}$−SH in ODE; from Parts b to f of Figure 1, the temperature was increased with the total elapsed time of 15 x 5 = 75 min. Here, the reaction was kept at 220 °C. Within 180 min, eight samples were extracted as indicated. For the measurement of optical absorption, an aliquot (20 µL) of each sample was dispersed in CH (3.0 mL). The absorption increased between 260 and 290 nm. The continuous increase of the sample absorption indicates the incessant formation of the PNC. (Scheme 1a Steps 1a/1b).

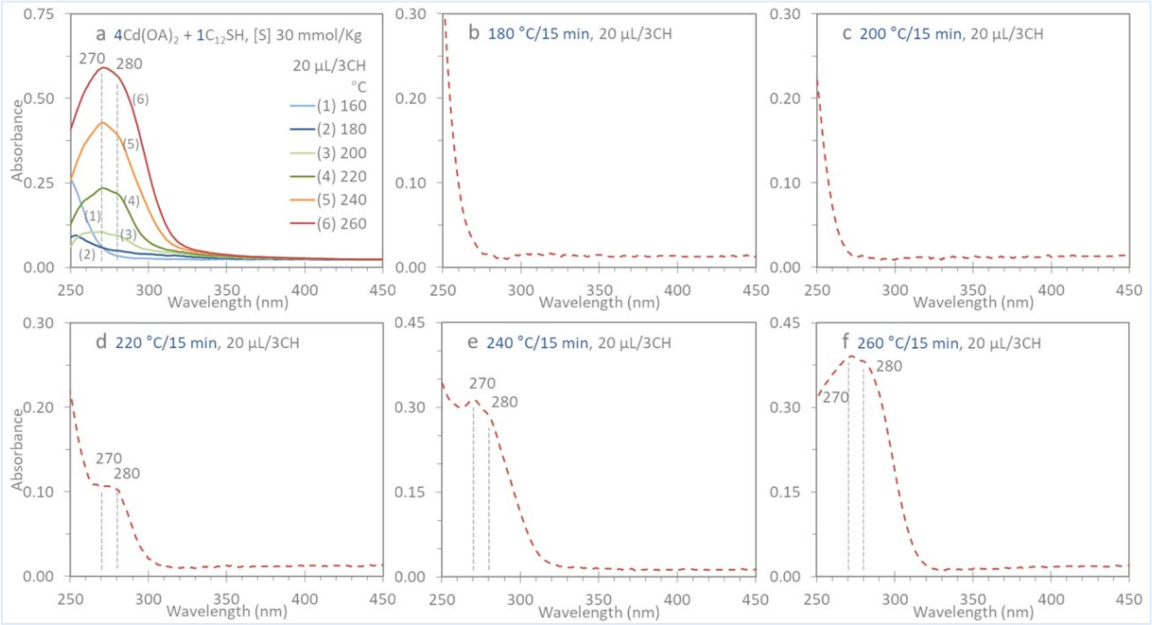

Figure S1-3A. Spectra after the background deduction. (a) The traces were obtained after the subtraction of the 140 °C trace in Figure 1a. (b–f) The dashed traces were obtained after the subtraction of the corresponding dashed traces in Parts b–f of Figure 1. The PNC displays a broad signal of optical absorption between 260 and 290 nm.

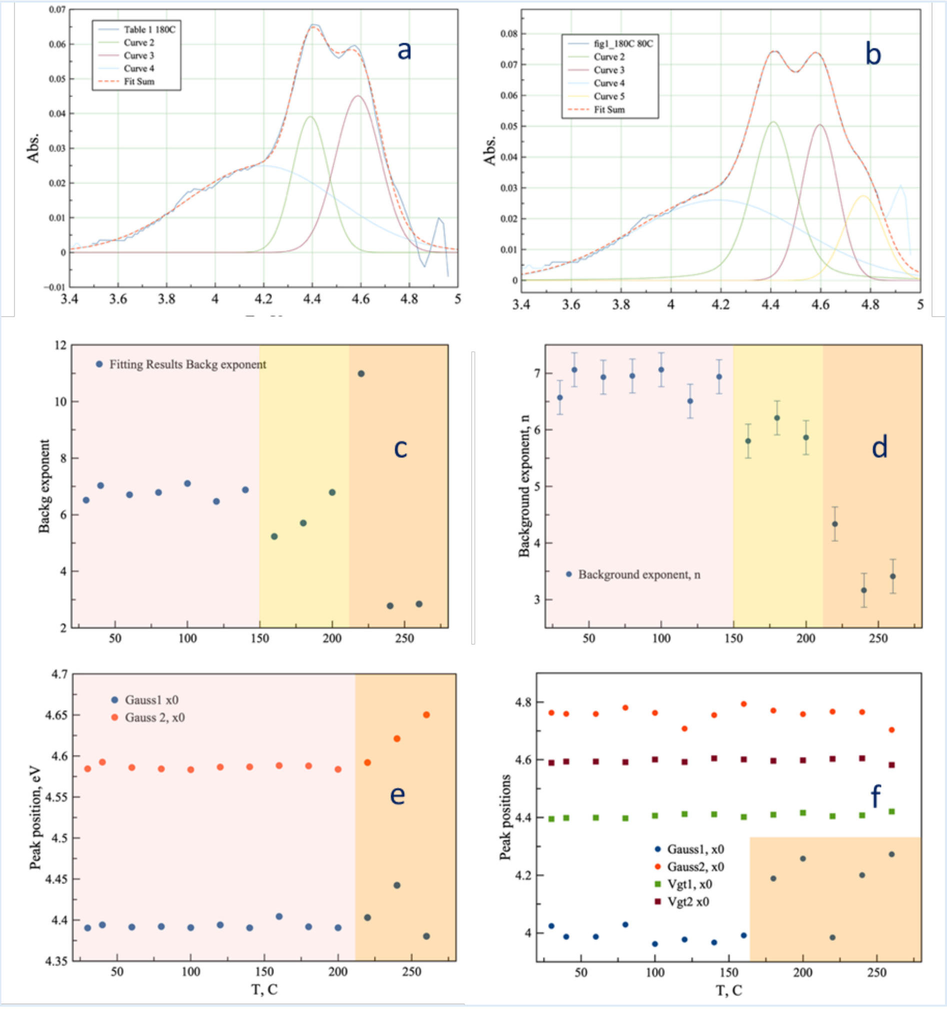

Figure S1-3B. The numerical analysis. This method is what we developed recently for the analysis of spectroscopic data.[1] The data for the Figure 1a sample traces (and for 30 and 40 °C samples) further supports the PNC formation, which can be seen in the evolution of the background exponent (how steep it is) and peak positions. The data were extracted by fitting the spectra in the energy space using two models with the common background exponential decay background function ($y = e^{(E-c)^n} + b$, where E is the energy and n, c, b are variables):

i) Gaussian profile to all peaks;

ii) pseudo-Voigt profiles to the peaks at 270 and 280 nm, Gaussian profiles to the rest of the spectrum.

Model ii), being more advanced, provides a noticeably better fit to the data (a, b). Comparison of the two models to gain insights into the evolution of the optical absorption spectra is shown. Both models show a change after 200 °C in the initially stable value of

background exponent n (c, d). The more advanced model ii) allows to localize the spectral changes largely to the evolution of a broad Gaussian peak at around 280 nm after ~180 °C (f, blue circles).

(1) Haddad, L.; Gianolio, D.; Sapelkin, A. Revisiting the Question of Information Content of EXAFS Spectra through a Bayesian Approach. https://arxiv.org/abs/2509.07950

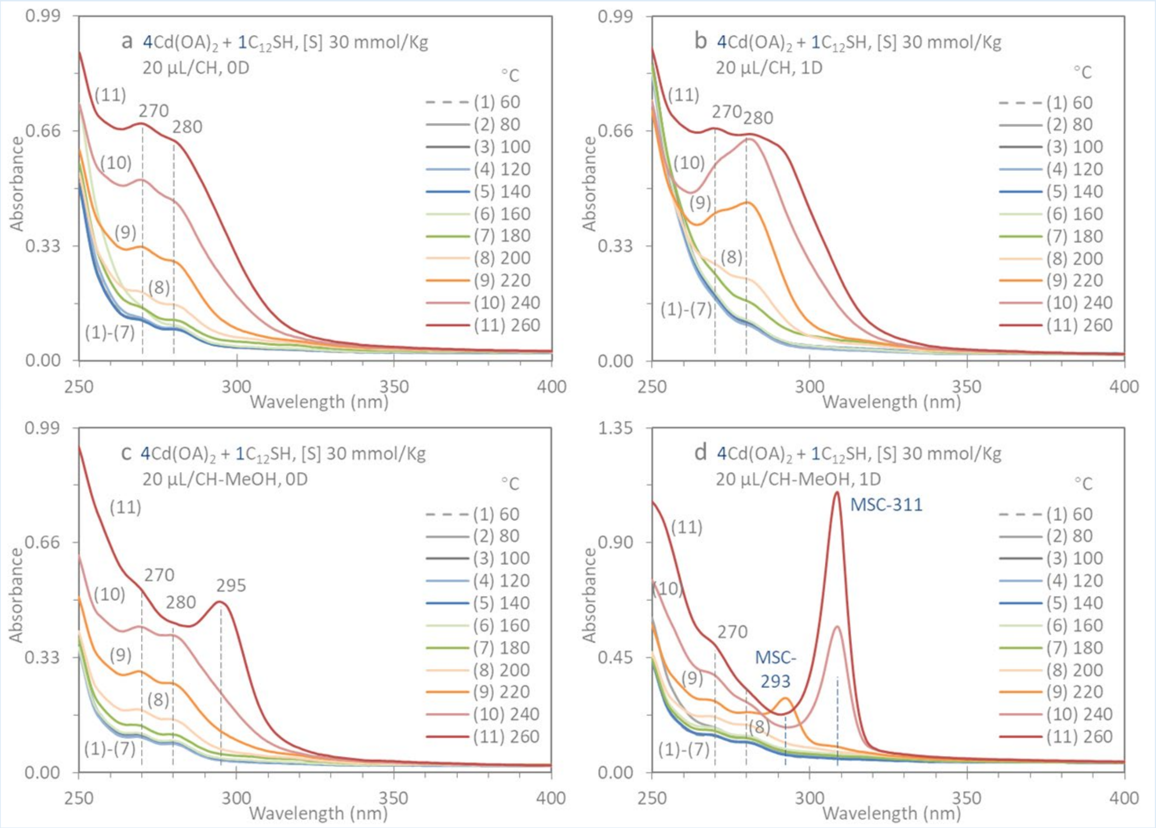

Figure S1-4. The four full sets of spectra for samples from the Figure 1a reaction. 11 samples were extracted as indicated. For the measurement of optical absorption, an aliquot (20 μL) of each sample was placed in CH (a, 0D) as well as in CH–MeOH (c, 0D). After 24 h (1D), the spectra were collected again which are shown in Parts b and d, respectively.

- When a sample (such as Sample 260 °C) is dispersed in CH (a trace 11) and in CH–MeOH (c trace 11), the absorption spectra can be quite different.
- (d) MSC-293 is seen for Sample 220 °C (trace 9), and MSC-311 for Samples 240 and 260 °C (traces 10 and 11). Our experimental observations indicate that MeOH accelerated the PNC–MSC isomerization. According to previous studies,[1-4] a process of ligand exchange

might take place as shown by the equations below.

MeO–H + RCOO–PNC → RCOO–H + MeO–PC-293

MeO–PC-293 → MeO–MSC-293

- In a side note, optical absorption spectroscopy is powerful in the detection of the appearance of a molecule. The process of optical absorption is associated with an electronic transition of the molecule, during which an electron of the molecule is elevated to a higher energy level. The non-covalent interaction (including H bonds) between two molecules may affect the spectra of a molecule to a certain degree, if the distribution of the electron cloud of the molecule changes. For the present study, only when the reaction temperature was above 200 °C (trace 8), the PNC formed with the Cd–S bond. When Samples 220–260 °C were placed in CH–MeOH after one day (d), MSC-293 and MSC-311 were seen due to the PNC isomerization.
- Based on the present reaction, the evidence of the PNC formation includes
  1. (a) For Samples obtained below 220 °C placed in CH (traces 1–8), the absorption strength between 260 and 290 nm increased little.
  2. (a) For Samples obtained above 220 °C placed in CH (traces 9–11), the absorption strength between 260 and 290 nm increased as the temperature increased.
  3. (d) When Samples obtained below 220 °C were placed in CH–MeOH after one day (traces 1–8), no MSCs were seen.
  4. (d) When Samples 220–260 °C were placed in CH–MeOH after one day (traces 9–11), MSC-293 and MSC-311 were seen due to the PNC isomerization.

(1) Hassinen, A.; Moreels, I.; De Nolf, K.; Smet, P. F.; Martins, J. C.; Hens, Z. Short-Chain Alcohols Strip X-Type Ligands and Quench the Luminescence of PbSe and CdSe Quantum Dots, Acetonitrile Does Not. *J. Am. Chem. Soc.* **2012**, *134*, 20705–20712.

(2) Zhu, T.; Zhang, B.; Zhang, J.; Lu, J.; Fan, H.; Rowell, N.; Ripmeester, J. A.; Han, S.; Yu, K. Two-Step Nucleation of CdS Magic-Size Nanocluster MSC-311. *Chem. Mater.* **2017**, *29*, 5727–5735.

(3) Yang, Y.; Huang, R.; Liu, Y.; Zhang, C.; Sapelkin, A. V.; Chen, X.; Yu, K. Monomer Substitution Assisted CdTeSe Magic-Size Cluster Development from CdTe and CdSe Prenucleation Clusters in Dispersion at Room Temperature. *Chem. Mater.* **2025**, *37*, 2855–2866.

(4) Su, J.; Gu, K.; Wang, Q.; Min, K.; Gao, Z.; Zhong, H. Alcohol Induced Surface Charging of Colloidal Quantum Dots for Controllable Electrophoretic Deposition Processing. *Nano Res.* **2026**, *19*, 94908059.

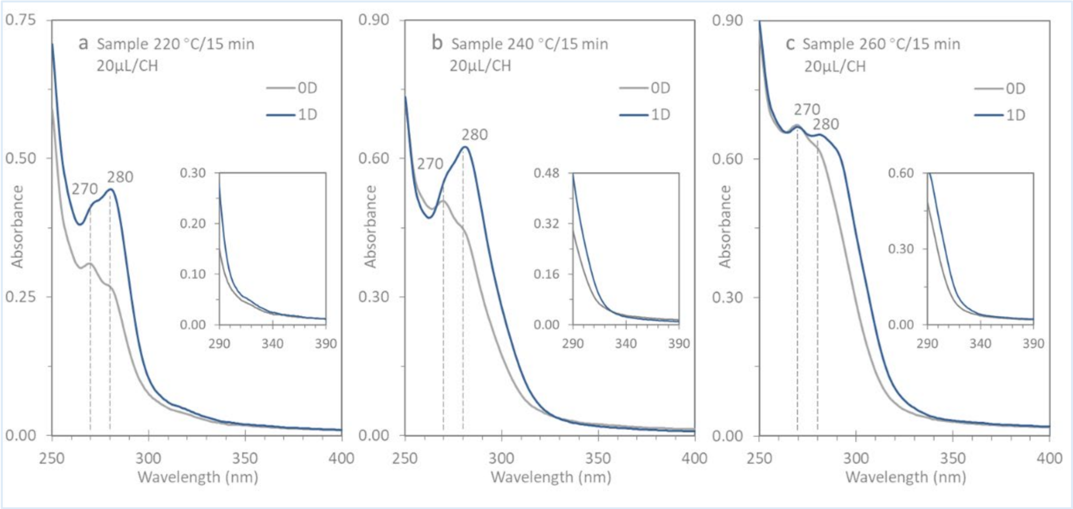

Figure S2-1. Comparison of sample absorption in dispersion of CH. The three samples were the Figure 2 samples taken at 220 °C (a), 240 °C (b), and 260 °C (c). Grey traces were collected immediately (0D), while blue ones after one day (1D). No MSCs developed after one day. Within 24 hours,

- from Samples 220 °C (a) to 240 °C (b), the absorbance at 280 nm increased more than that at 270 nm
- Sample 260 °C (c), the absorbance at 270 nm changed little while that at 280 nm increased accompanied by the appearance of a bump at ~290 nm.

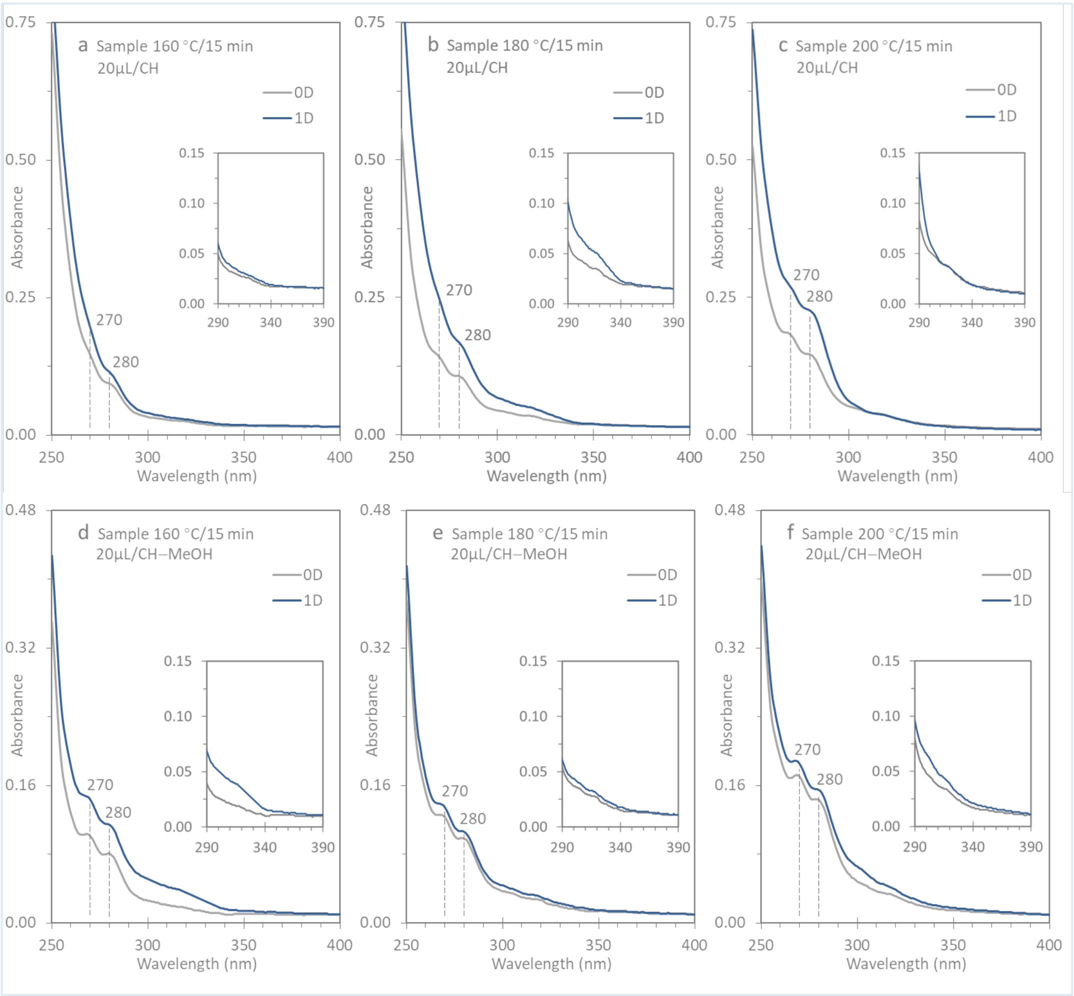

Figure S2-2. Comparison of sample absorption in dispersion. The three samples are the Figure 1a samples taken at 160 °C (a and d), 180 °C (b and e), and 200 °C (c and f). The samples were dispersed in CH (top panel Parts a–c) and in CH–MeOH (bottom panel Parts d–f). Grey traces were collected immediately (0D), while blue ones after one day (1D). After one day, no MSCs were seen.

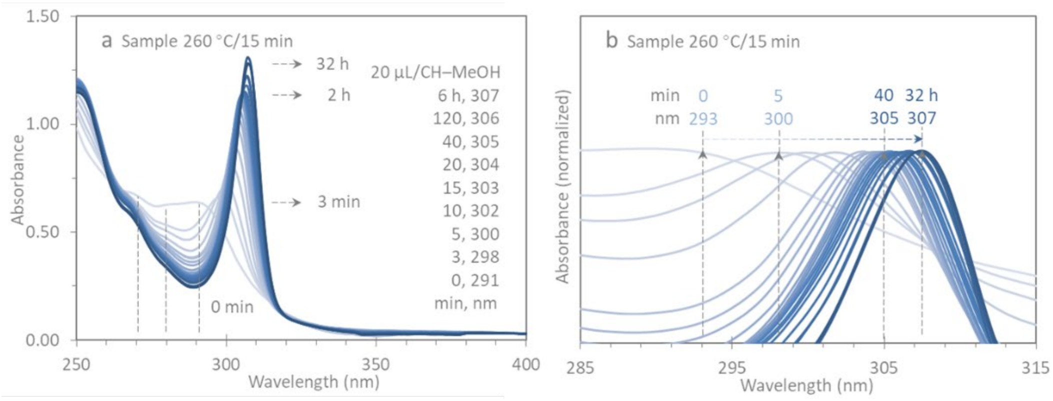

Figure S2-3. The full set of spectra for Parts d and e of Figure 2. 23 spectra were collected within 32 h. The spectra collected at 0, 5, and 40 min in Part b are indicated, together with that at 32 h.

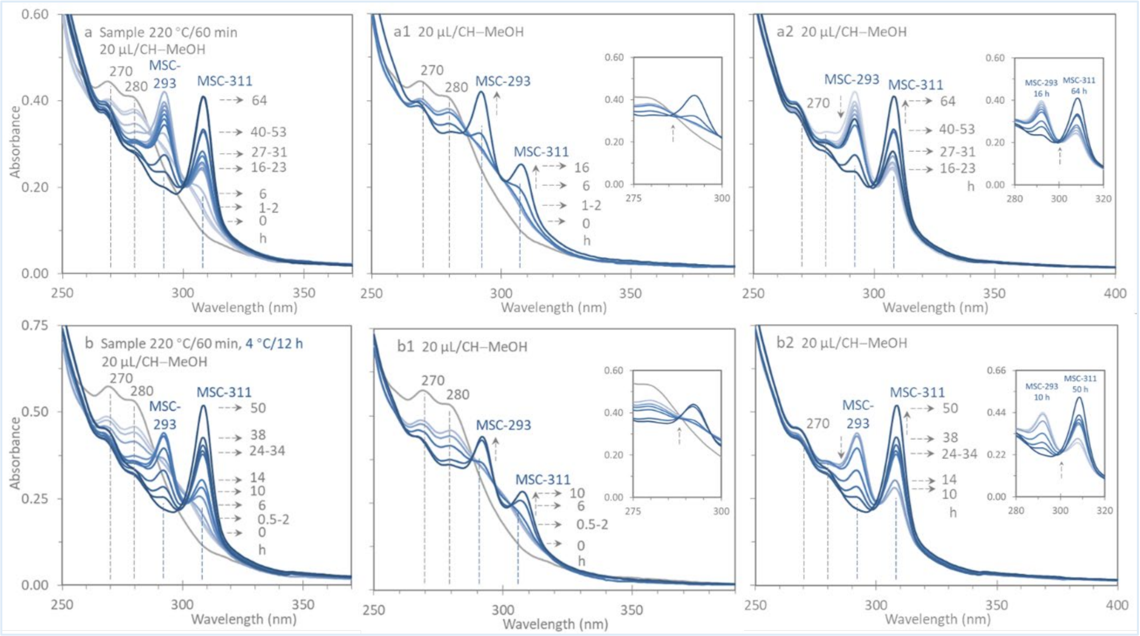

Figure S3-1. Isomerization of the CdS PNC (220 °C/60 min) in dispersion. Sample 220 °C/60 min was from the Figure 3 batch. The presentation format is similar to that of Figure 3. Similar results were obtained. A Figure 1a reaction was kept at 220 °C, and four samples were taken after 60, 90, 120, and 150 min. The last two samples are shown in Figure S3-2.

- For the measurement of optical absorption, an aliquot (20 µL) of the sample (without storage) was placed in CH–MeOH. 13 spectra were obtained within 64 h (a); they are presented in Parts a1 and a2. Part a1 has the first five spectra (0–16 h), and Part a2 has 9 spectra (16–64 h).
- For the measurement of optical absorption, the sample (20 µL) was incubated at 4 °C for 12 h, and CH–MeOH was added to the sample. The resulting dispersion was placed into a cuvette. 12 spectra were obtained within 50 h (b); they are shown in Parts b1 and b2. Part b1 has the first six spectra (0–10 h). Part b2 has seven spectra (10–50 h).

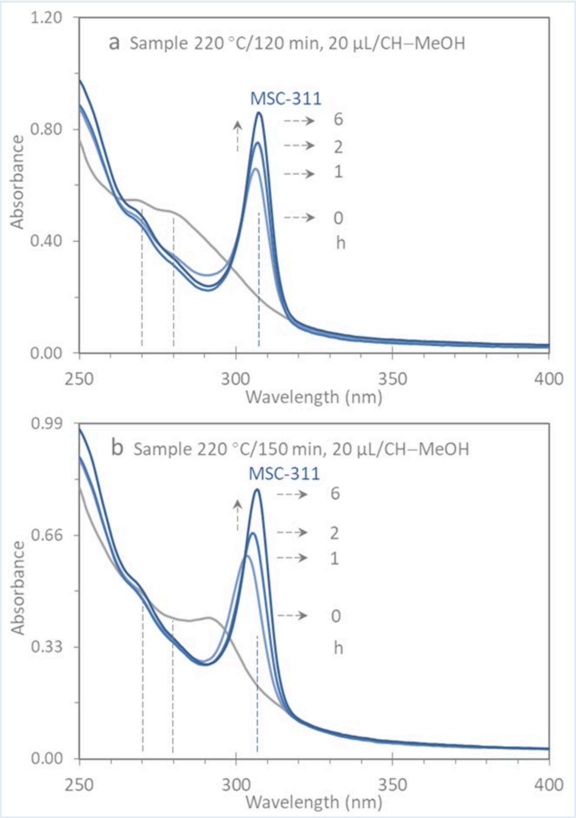

Figure S3-2. Isomerization in CH–MeOH of the CdS PNC in Sample 220 °C/120 min) (a) and Sample 220 °C/150 min (b). The two samples were from the Figure 3 batch. Each (20 µL) was placed in CH–MeOH.

- Within 1 h, the PNC isomerized to MSC-311 (Scheme 1b Steps 1/3/4); the strength at 270–280 nm decreased. After 1 h, the strength at 270–280 nm changed little, while that at ~310 nm kept increasing (within 6 h).
- The development pathway of MSC-311 had the direct transformation from MSC-293 (Scheme 1b Step 5), as well as via Scheme 1b Steps 3/4 from PC-293. This was explained in more detail by Parts d and e of Figure 2.
- MSC-311 is more ready to show up (than MSC-293) when a relatively late-stage sample is placed in CH–MeOH. In a reaction, the transformation from the PNC to PC-293 and to PC-311 may take place, and the energy barrier might increase slightly from the PNC to PC-293 and then to PC-311.[1]

(1) Wang, T.; Wang, Z.; Wang, S.; Chen, X.; Luan, C.; Yu, K. Thermally-Induced Isomerization of Prenucleation Clusters During the Prenucleation Stage of CdTe Quantum Dots. *Angew. Chem. Int. Ed.* **2023**, *62*, e202310234.

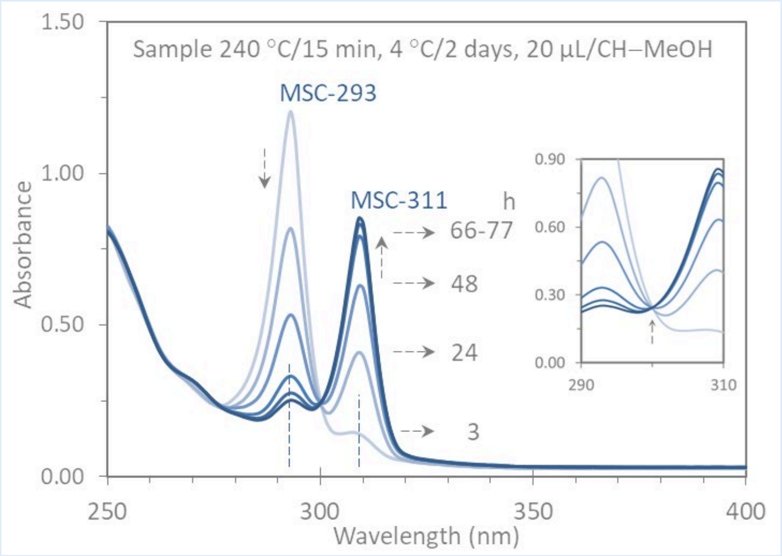

Figure S4-1. Isomerization from MSC-293 to MSC-311 in dispersion. Sample 240 °C/15 min was used for Part b of Figure 4; here, it was stored at 4 °C for two days before in CH–MeOH.

- For the measurement of optical absorption, 20 µL of the incubated sample was placed into a room-temperature mixture of CH–MeOH. Six spectra were collected from 3–77 h as indicated. MSC-293 and MSC-311 developed within 3 h; afterwards, MSC-293 transformed to MSC-311.
- An isosbestic point located at 300 nm. The relatively small change in the strength of the range of 250–275 nm suggests little involvement of the PNC during the MSC-293 to MSC-311 transformation (Scheme 1b Steps 2'/3/4).

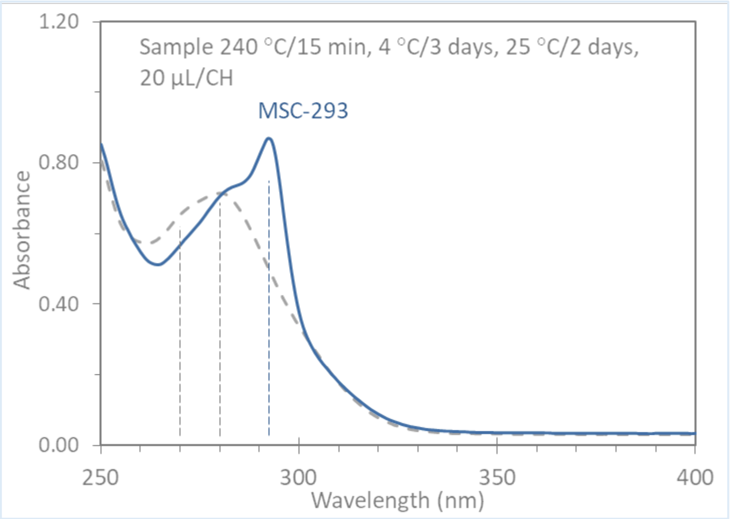

Figure S4-2. The Part b of Figure 4 sample stored at 4 °C for 3 days and at 25 °C for 2 days. For the measurement of optical absorption, 20 μL of the sample without and with incubation was added to CH (3.00 mL). The dashed grey trace is for the former, while the solid blue trace for the latter. MSC-293 was seen (blue trace) (Scheme 1b Steps 1/2). During the five-day storage, the PNC isomerized to PC/MSC-293 mainly (Scheme 1b Steps 1/2).

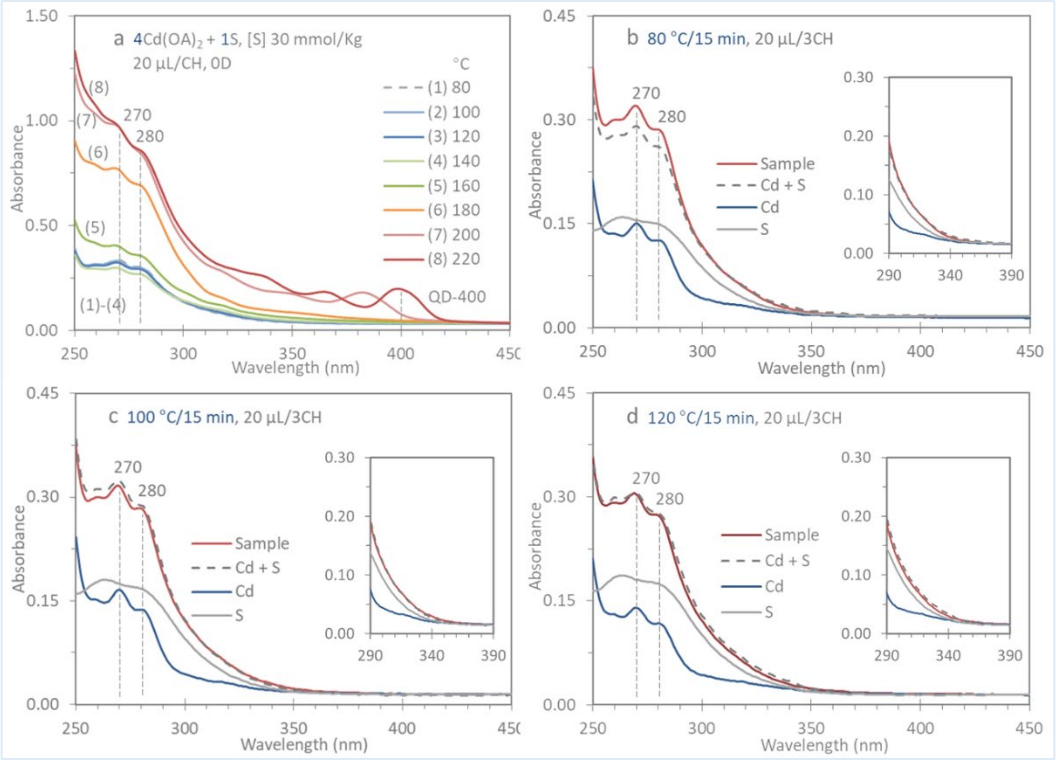

Figure S5-1. Development of the CdS PNC above 160 °C in the reaction of $Cd(OA)_2$ and S in ODE. The presentation format is similar to that of Figure 5, with the full set of the absorption spectra of the eight samples in Part a. Absorption comparison for the early-stage samples of 80 °C (b), 100 °C (c), and 120 °C (d) is similar to Parts b–f of Figure 5.

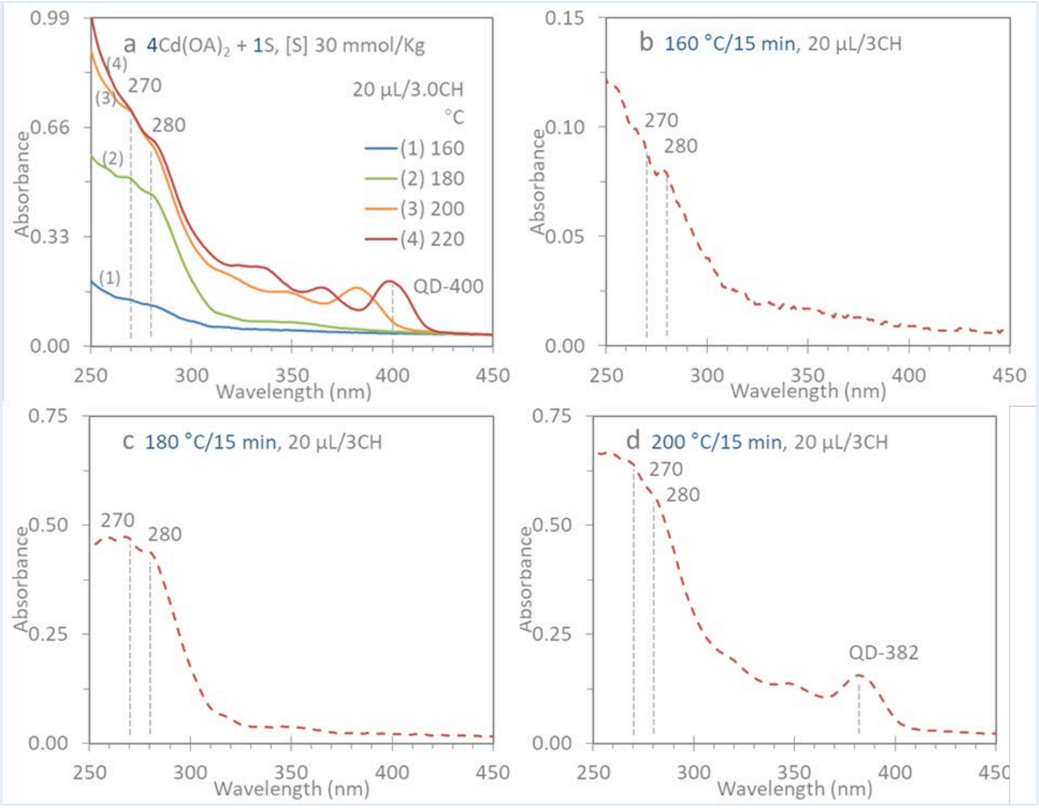

Figure S5-2. Spectra after the background deduction. (a) The traces were obtained after the subtraction of the 140 °C trace in Figure 5a. (b–d) The dashed traces were obtained after the subtraction of the corresponding dashed traces in Parts c–e of Figure 5. The PNC displays a broad signal of optical absorption between 260 and 290 nm.

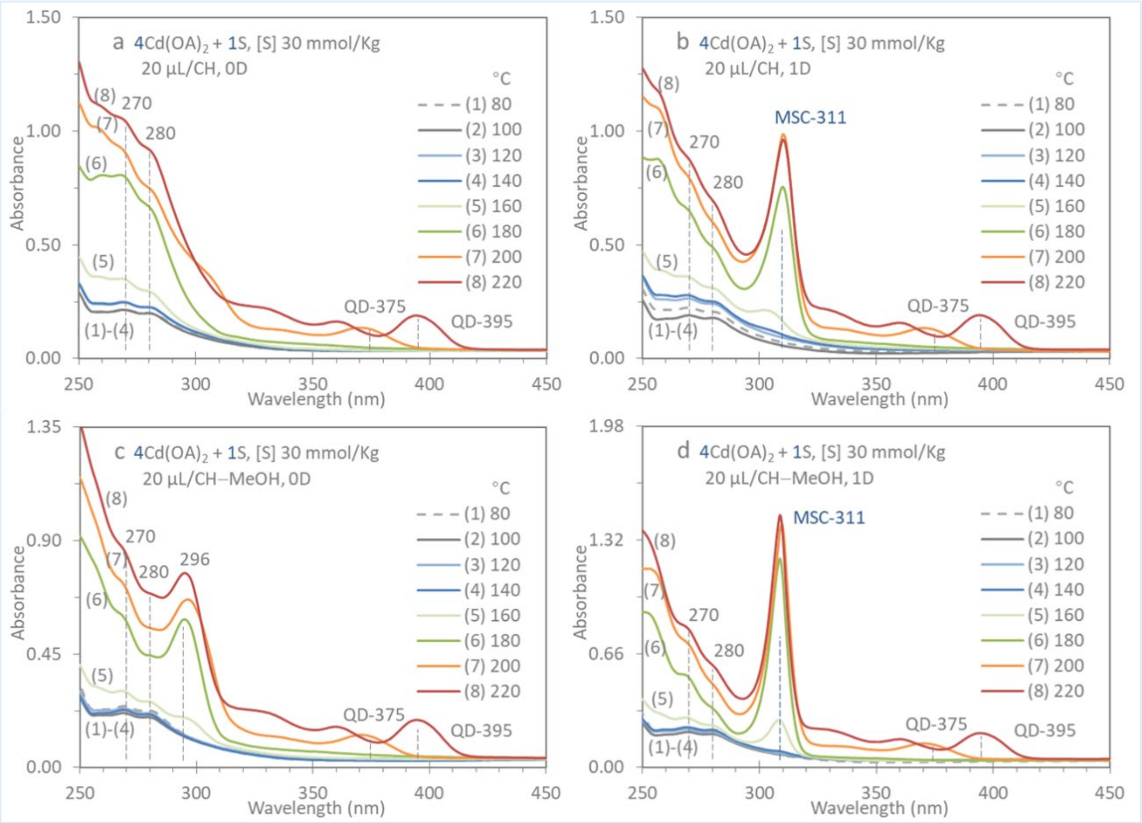

Figure S5-3. The four full sets of spectra of the samples from a different Figure 5 reaction batch. Eight samples were extracted as indicated. For the measurement of optical absorption, an aliquot (20 μL) of each sample was placed in 3.0 mL of CH (3CH) (a 0D and b 1D) as well as in CH–MeOH (c 0D and d 1D). Samples 200 °C and 220 °C had QD-375 and QD-395, respectively, together with the PNC.

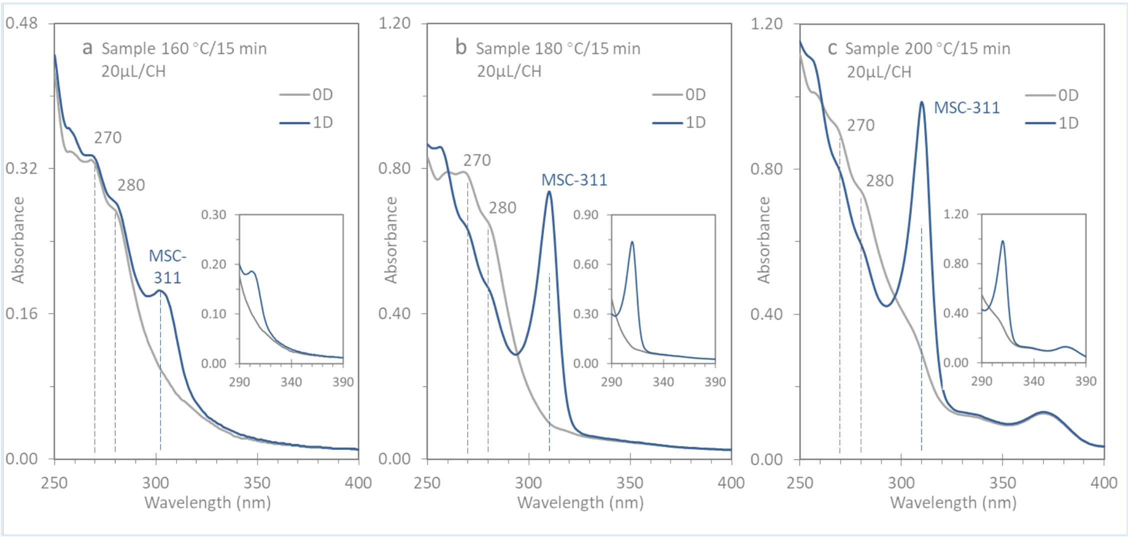

Figure S6-1. Comparison of sample absorption after one day in dispersion. The three samples are the Figure S5-3 samples taken at 160 °C (a), 180 °C (b), and 200 °C (c). Grey traces were collected immediately, while blue ones were collected after one day. After one day in CH, MSC-311 was seen.

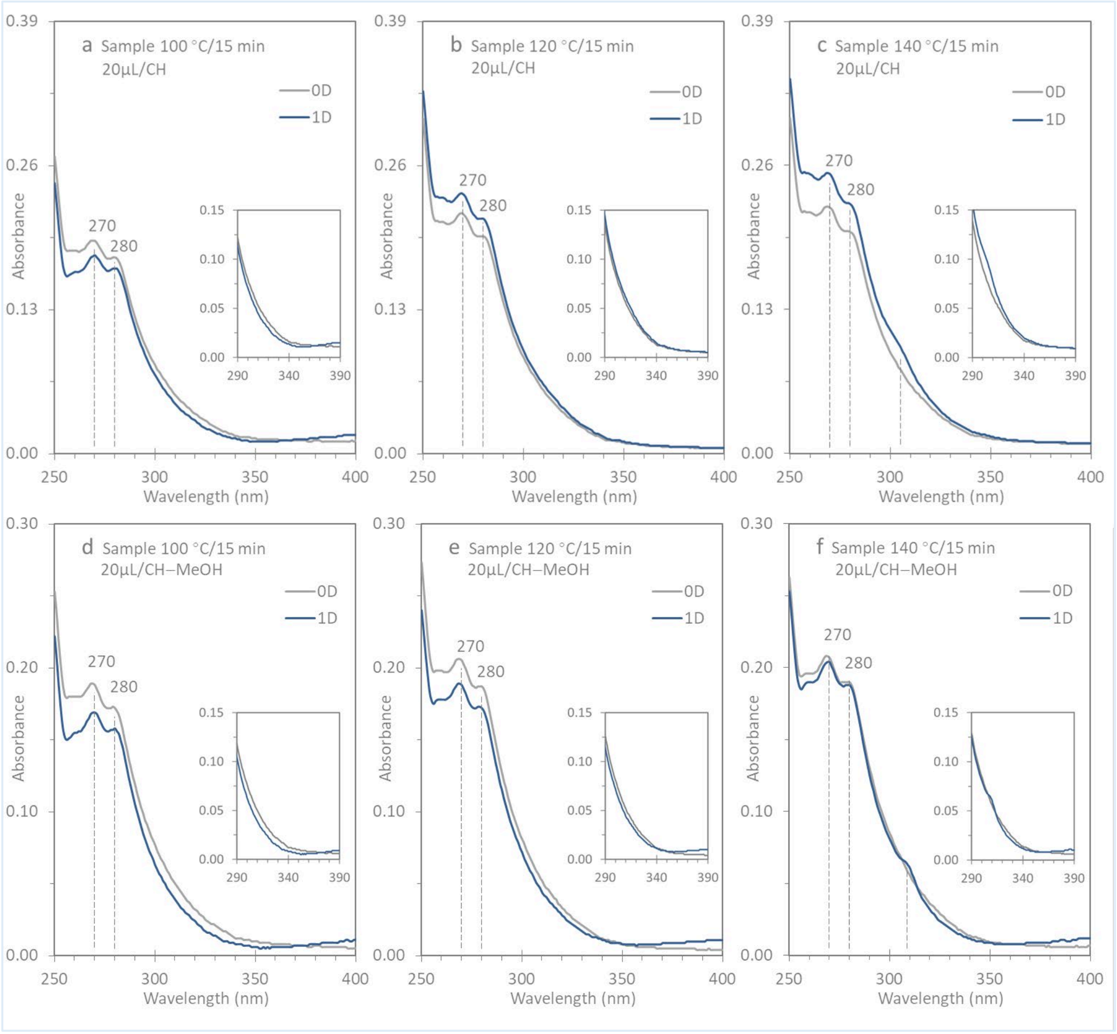

Figure S6-2. Comparison of sample absorption in dispersion. The three samples are the Figure S5-3 samples taken at 100 °C (a and d), 120 °C (b and e), and 140 °C (c and f). The samples were dispersed in CH (top panel Parts a–c) and in CH–MeOH (bottom panel Parts d–f). Grey traces were collected immediately (0D), while blue ones after one day (1D). After one day, no MSCs were seen, with a negligible amount of MSC-311 in Parts c and f.

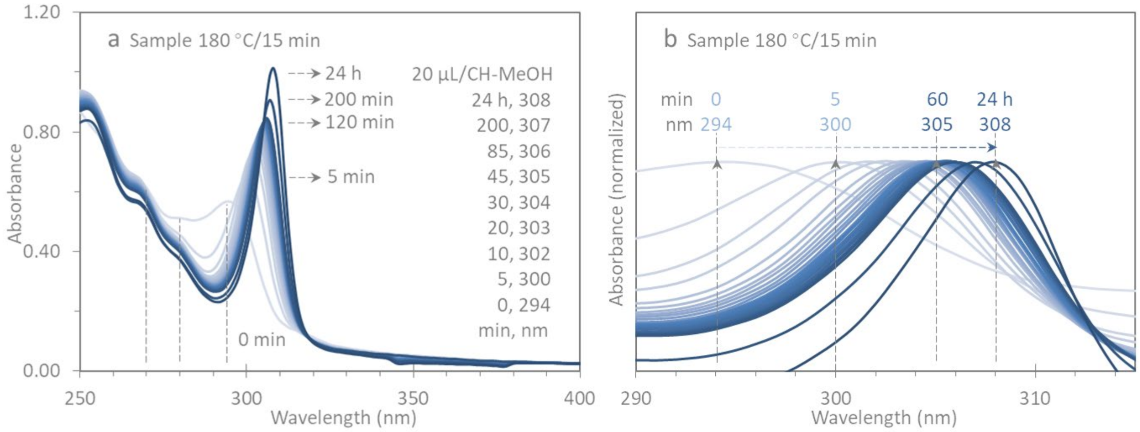

Figure S6-3. The full set of optical absorption spectra of for Parts d and e of Figure 6. There are 27 spectra collected within 24 h.
